# Photocatalytic proton reduction with ruthenium and cobalt complexes immobilized on fumed reversed-phase silica†
†Electronic supplementary information (ESI) available. See DOI: 10.1039/c5sc02124c


**DOI:** 10.1039/c5sc02124c

**Published:** 2015-10-08

**Authors:** C. Bachmann, B. Probst, M. Oberholzer, T. Fox, R. Alberto

**Affiliations:** a Department of Chemistry , University of Zürich , Winterthurerstr. 190 , CH-8057 Zürich , Switzerland . Email: ariel@chem.uzh.ch

## Abstract

Heterogeneous photocatalytic hydrogen production with a non-covalently immobilized molecular ruthenium based photosensitizer (PS) and a cobalt polypyridyl based water reducing catalyst (WRC) is reported. PS and WRC were derivatized with C_18_-alkyl chains and immobilized by adsorption on hydrophobic fumed silica. The resulting loaded support was suspended in water with anionic or cationic surfactants and subjected to heterogeneous photocatalytic H_2_ production with ascorbate as sacrificial electron donor (SED). No leaching was observed under catalytic conditions, thus catalysis was truly heterogeneous. The catalytic performance of immobilized PS and WRC clearly exceeded that of homogeneous catalysis at low concentrations. At high concentration, diffusion and light limitation lead to lower reaction rates, but the same stability as for homogeneous reactions was still achieved. WRC concentration variations indicated a relatively high stability (up to 1300 H_2_/Co) and mobility of amphiphilic catalysts on the hydrophobic silica surface. Comparison of fumed silica with porous and non-porous silica showed, that a high BET surface area along with a good accessibility from the reaction media are crucial for catalytic performance. Mechanistic investigations by transient absorption spectroscopy displayed reductive quenching of excited PS by ascorbate followed by on particle electron transfer to WRC as reaction pathway. Particles with additional cationic surfactants exhibited a significantly higher catalytic performance as compared to anionic surfactants. Non-covalent anchoring of correspondingly derivatized WRCs or PSs to reversed-phase silica offers a rapid and versatile transition from homogeneous to heterogeneous molecular proton reduction.

## Abstract 2

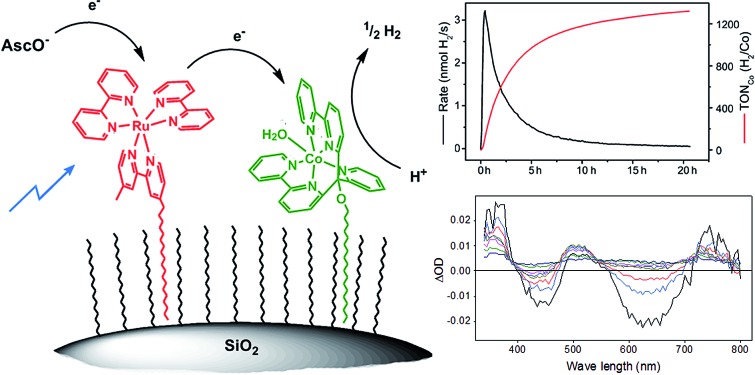
Non-covalent binding of water reduction catalysts and photosensitizers to hydrophobic silica particles represents a general approach for heterogenizing molecular water splitting components.

## Introduction

Photocatalytic water splitting into H_2_ and O_2_ with sun light (artificial photosynthesis) is a promising way to store solar energy in chemical bonds.^1^–^3^ To drive the two half reactions, water oxidation and reduction, of this highly complex process with artificial molecular catalysts or (nano)materials remains a challenge. Approaches based on purely molecular components are complemented by strategies relying on materials.^4^–^6^ Over the last decade, active, molecular water oxidizing and reducing catalysts (WOC, WRC) have been developed,^2^,^7^–^15^ (and references therein) but light driven, full water splitting was mainly achieved with semiconductors or dye sensitized materials.^5^,^16^,^17^ A fully homogeneous, molecular water splitting architecture is unlikely to exist since numerous back and cross electron transfers (shortcuts) between the O_2_ and H_2_ evolving half reactions (OER, HER) lead to self-inhibition.^8^ Molecular (photo)catalysts for both reactions, immobilized on nanomaterials in the same solution and connected through an electron relay, have been proposed as a viable route towards “one pot” water splitting, despite the disadvantage of getting mixtures of O_2_ and H_2_.^18^ Molecular catalysis on small solid phase particles is a topic of intensive research. Various methods, including covalent and non-covalent linking of molecular catalysts on polymeric or inorganic supports have been described for synthetic purposes.^19^–^26^ Covalently bound catalysts are less prone to leaching, but specific synthetic strategies are required, which limit screening and reduce flexibility with respect to catalysts. Non-covalent immobilization – mostly by encapsulation or ionic, polar and apolar adsorption – is more convenient but weaker interactions lead to increased catalyst release and thus restricted applications.^20^,^22^–^25^ Studies with particle bound, pure molecular catalysts for photocatalytic water oxidation or reduction are relatively rare. Particular examples rely on functional supports such as semiconductors, electrodes or quantum dots covered with molecular WOCs or WRCs.^27^–^40^ Reisner and co-workers chemisorbed Co and Ru catalysts by polar interactions on TiO_2_ and ZrO_2_ particles. They observed “through particle” electron transfer (ET) for TiO_2_ and “on particle” ET for ZrO_2_.^41^,^42^ Meyer and co-workers recently reported a procedure to electropolymerize Ru based PS and WOC on TiO_2_ particles for photo- and electrocatalytically active electrodes.^43^ Inspired by nature, König, Sun and co-workers embedded alkylated Co and Ru complexes in phospholipid membranes and observed a remarkable activity for both – oxidative and reductive – half reactions.^44^,^45^ Anchoring in these membranes has the advantage of molecular mobility on the carrier material but the disadvantage of chemical and physical instability whereas the oxide materials required particular anchoring groups.

Silica particles, coated with hydrophobic, long alkyl chains, represent a combination between oxidic nanoparticles and membranes. These so-called reversed-phase materials as used in HPLC and preparative column chromatography are chemically and physically inert and interact strongly with molecules comprising pendent lipophilic groups. These strong, non-covalent interactions suppress leaching while keeping mobility on the alkylated surface intact. With minimal derivatization, essentially any catalyst or photosensitizer (or both) can be anchored on these materials. Hydrophobic interactions^46^ were widely investigated for protein immobilization and synthetic purposes.^22^,^23^,^47^ Adsorption by distinct alkyl–alkyl interactions are, however, rare; one particular example is based on fluorocarbon – derivatized catalysts adsorbed on fluorous reverse phase silica for Pd catalyzed cross coupling reactions.^22^

**Scheme 1:**
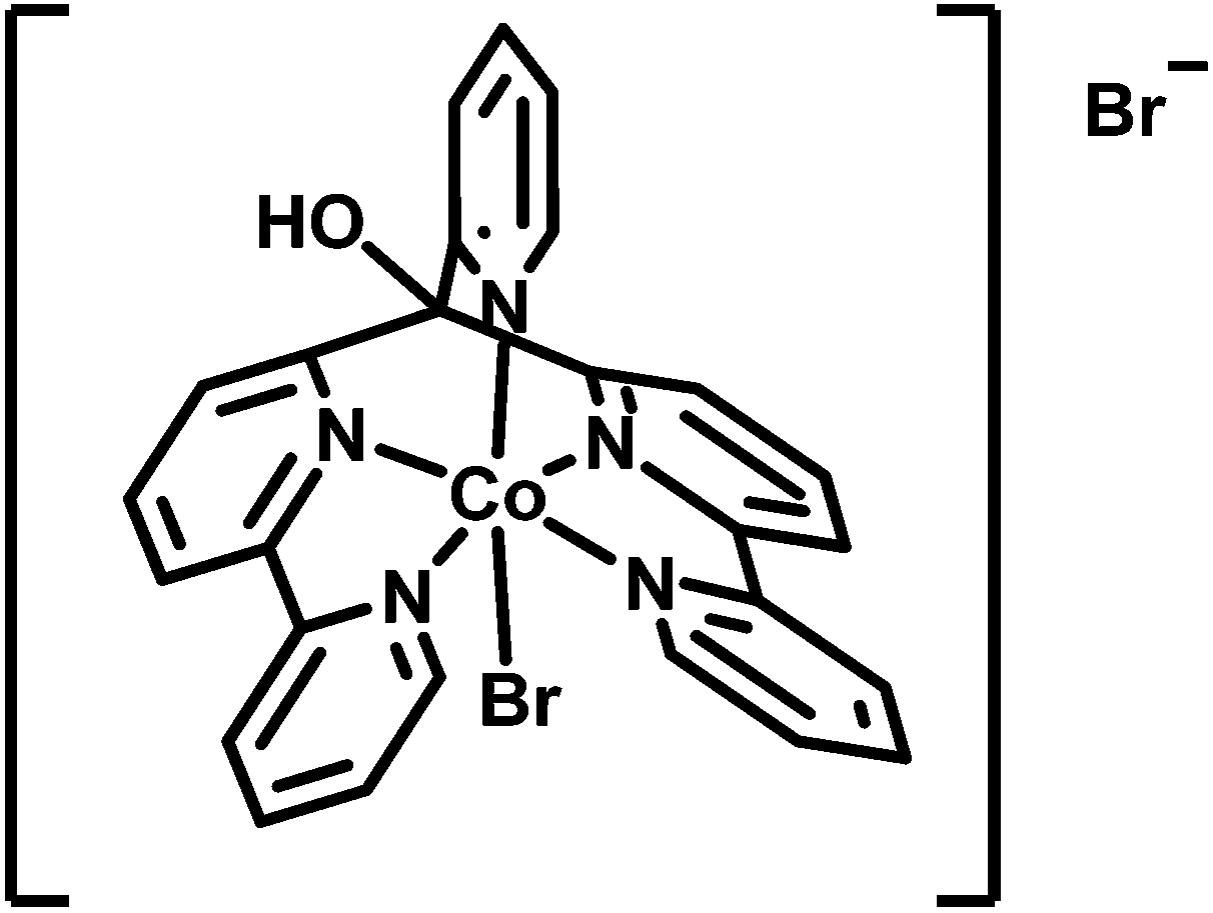
Structure of water reducing catalyst [Co^II^Br(appy)]Br **1**.^52^,^54^

Acyclic cobalt complexes with poly-pyridyl ligands are a focus of recent research.^14^,^48^–^56^ In our studies, the complex [Co^II^Br(appy)]Br **1** together with Re or Ru based PSs exhibited excellent proton reducing properties in homogeneous aqueous solution (Scheme 1).^52^,^54^ A convenient way to immobilize these highly active WRCs alone or together with appropriate PSs on a robust support represents an important step towards a heterogenized architecture with molecular catalysts. Aforementioned non-covalent anchoring on solid phase materials *via* hydrophobic adsorption is straightforward and displacement of the components in aqueous media by protonation or competing (ionic or polar) species are greatly diminished.

We present in this study a flexible method to immobilize WRC **1** and [Ru(bpy)_3_]Cl_2_, both derivatized with long alkyl chains, on hydrophobic fumed silica particles, a cheap, robust and commercially available high BET surface area material. As fumed silica is non-porous with high accessibility, good catalytic performance was expected from these composites compared to other silica based particles. For comparison, the catalysts were also anchored on porous silica with very high BET surface area but low accessibility and on non-porous, spherical silica with high accessibility but low BET surface area. These nanocomposites are applied to heterogeneous, photocatalytic proton reduction and efficiency is compared to catalysis in homogeneous solution under equal conditions. The concept of anchoring catalysts on the hydrophobic surface of nano- or micromaterials is an excellent way of assessing the long-term performance of molecular systems.

## Results and discussion

### Synthesis and immobilization

**Scheme 2:**
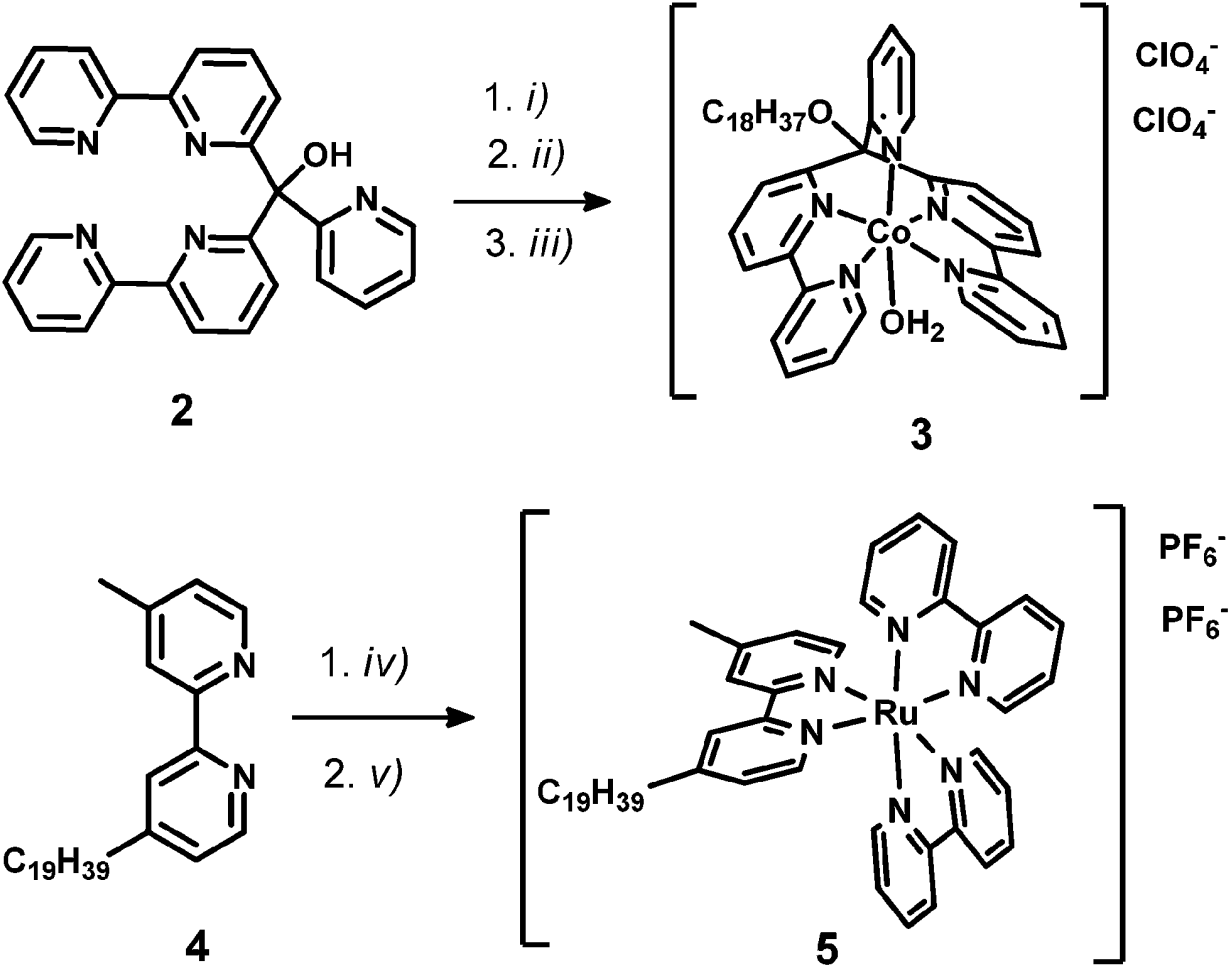
Schematic representations of syntheses towards C_18_/C_19_-derivatized WRC and PS: (i) NaH, DMF, rt, 45 min; (ii) C_18_H_37_-I, rt, 15 h; (iii) Co(ClO_4_)_2_, MeOH, rt, 3 h; (iv) Ru(bpy)_2_Cl_2_, EtOH/H_2_O, 100 °C, 24 h; (v) NH_4_PF_6_, H_2_O. Detailed synthetic procedures are given in the experimental part.

WRC [CoBr(C_18_-appy)]Br (**3**) was synthesized in two steps; first, the hydroxy group of the basic polypyridyl ligand framework **2** (appy) was alkylated with octadecyliodide, then WRC **3** was obtained by coordination to Co^II^ in 79% overall yield. The alkylated bpy ligand 4-methyl-4′-nonadecyl-2,2′-bipyridine (C_19_-bpy, **4**) was obtained by deprotonation of 4,4′-dimethyl-2,2′-bipyridyl with Li[N(isopropyl)_2_] (LDA) followed by reaction with 1-bromooctadecane. The PS [Ru(bpy)_2_(C_19_-bpy)]^2+^ (**5**) finally was prepared from *cis*-[Ru(bpy)_2_Cl_2_] with **4** and subsequent counter-ion exchange with NH_4_PF_6_ (Scheme 2).

**Scheme 3:**
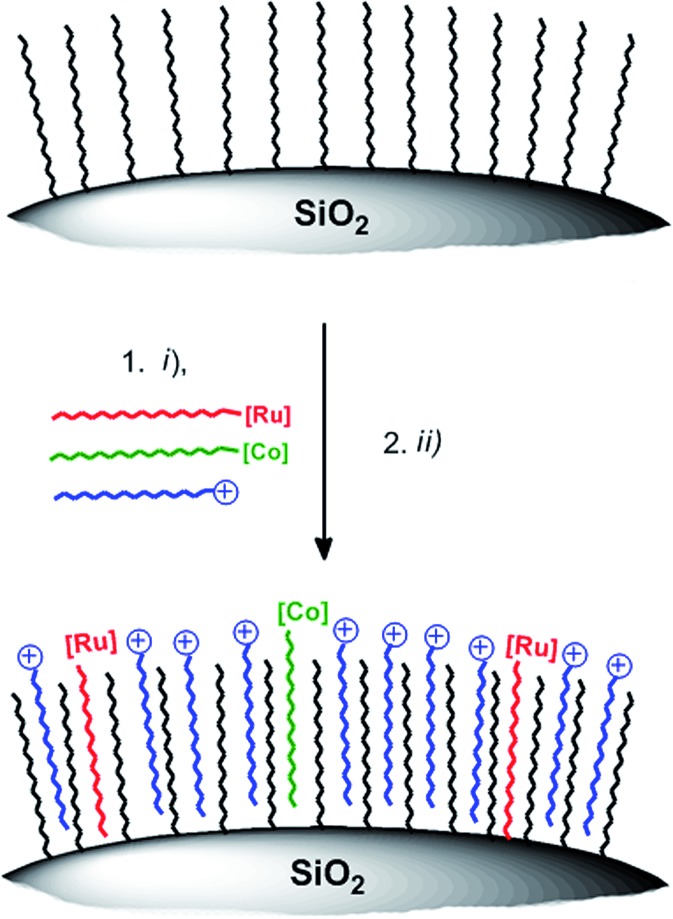
Schematic illustration of WRC and PS adsorption on hydrophobic silica. (i) **3**, **5**, **7** (or **6**), MeOH, rt, 30 min (ii) 0.1 M NaOTf electrolyte, MeOH evaporation. Detailed synthetic procedures are given in the experimental part.

Stirring hydrophilic fumed silica particles (f-SiO_2_) in CH_2_Cl_2_ containing octadecyltrichlorosilane gave the hydrophobic fumed silica support f-SiO_2_-C_18_. The support was then loaded with 0.15 μmol WRC and PS (**3** and **5**) per m^2^ BET surface area (equals 0.1 molecule per nm^2^, ∼3 mass% for fumed silica). To reduce particle agglomeration in aqueous solution and to increase the surface hydrophilicity, the surfactants sodium 4-dodecylbenzenesulfonate (Na[C_12_-PhSO_3_], **6**) or *N*,*N*,*N*-trimethylhexadecyl ammonium acetate ([C_16_-NMe_3_][OAc], **7**, Scheme SI1†) were co-loaded. Stirring of respective methanol (MeOH) solutions in the presence of f-SiO_2_-C_18_, followed by the addition of an aqueous electrolyte and subsequent MeOH evaporation gave orange and luminescent silica particles which were isolated by centrifugation or filtration. Together with amphiphiles **6** and **7**, the formation of a mono-layer arrangement as shown by Ducker *et al.* for different ionic and non-ionic surfactants (Scheme 3) is expected.^57^ Quantitative loading was assessed by HPLC analysis: the absence of a complex peak in the supernatant confirmed complete adsorption **3** and **5**, on f-SiO_2_-C_18_.

Particles loaded with complex **5** were examined by ^13^C-CPMAS solid state NMR spectroscopy and compared to pure **5** as solid or in solution. The determined signals with loaded silica can clearly be assigned to adsorbed PS **5** (Fig. SI1†).

**Fig. 1:**
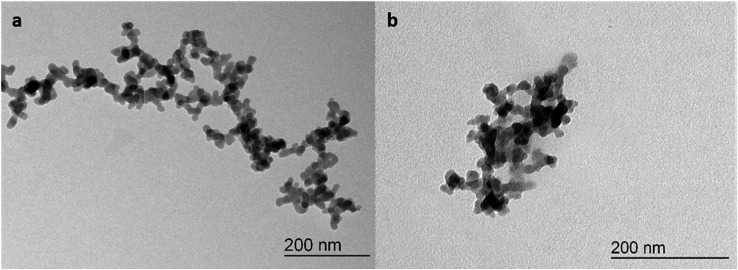
Representative TEM micrograph of hydrophilic fumed silica without (a) and hydrophobic fumed silica with adsorbed PS **5** from a diluted aqueous suspension with **7** as surfactant (b).

Transmission electron microscopy measurements (TEM, Fig. 1) of f-SiO_2_ before and after silylation and loading with **5** clearly showed that the structure of fumed silica is not modified neither by silylation nor by double layer formation with the adsorbents. The particles still consist of *ca.* 20 nm spheres, which are condensed to chains and branches, forming particles of 200–500 nm in diameter. DLS measurements of these silica suspensions in water (at 1/1000 dilution) were in agreement with TEM results, and mean hydrodynamic diameters of several 100 μm were found both for unloaded and loaded f-SiO_2_-C_18_ (Table SI1†). A strong particle size dependency on the surfactant concentration was found. Low amounts of surfactant (<1 mM) did not fully suppress aggregation (Fig. SI2†) whereas too high amounts of amphiphiles (>10 mM) lead to release of adsorbed WRC or PS from the surface. Exposition and loading with 1–4 mM surfactant solutions were optimal for separating the particles to a homogeneous suspension.

Equally important as the surface hydrophilicity of the particles is the ionic strength of the electrolyte solution. Enhancing the ionic strength of solutions with suspended particles reduces the electrical double layer thickness around the nanoparticles. Repulsive forces are thus reduced and aggregation is increased.^58^–^60^ Indeed, addition of NaOTf electrolyte (0.1 M), as used in the photocatalysis experiments (*vide infra*), lead to a significantly increased particle size and size distribution (*d* = 0.5–5 μm, Table SI1†). Ascorbate buffer also leads to an increased hydrodynamic diameter, but the effect was clearly lower as compared to NaOTf. As DLS and TEM measurements demand high dilutions, suspensions at catalytic concentrations (*ca.* 7–8 mg per mL reaction solution) were studied by fluorescence microscopy (FM) using f-SiO_2_-C_18_ loaded with PS **5**. Under these conditions, aggregates ranging from <1 μm (detection limit) up to 50 μm were observed (Fig. SI3†), possibly because of coagulation due to sedimentation in the measurement void between the two glass plates used for fluorescence microscopy. As shown in Fig. SI4,† dye **5** is evenly distributed in the aggregates, in line with the notation off small, dye decorated particles that aggregate.

### Photocatalysis

The loading processes on f-SiO_2_-C_18_ particles as described before enable a convenient variation of the WRC/PS ratios, their absolute concentrations and the nature of the surfactants, respectively. WRC **3** and PS **5** were bound to the particles and amphiphile **7** was added to increase wettability. The photocatalytic H_2_ evolution experiments were performed in 1 M ascorbic acid/ascorbate buffer (H_2 asc_/H_asc_^–^, pH = 4) as SED and with 0.1 M NaOTf as inert electrolyte. PS and WRC concentrations in these experiments are defined as the total amount (in mol) of immobilized complex, grafted on the support per catalysis solution volume (10 mL).

**Fig. 2:**
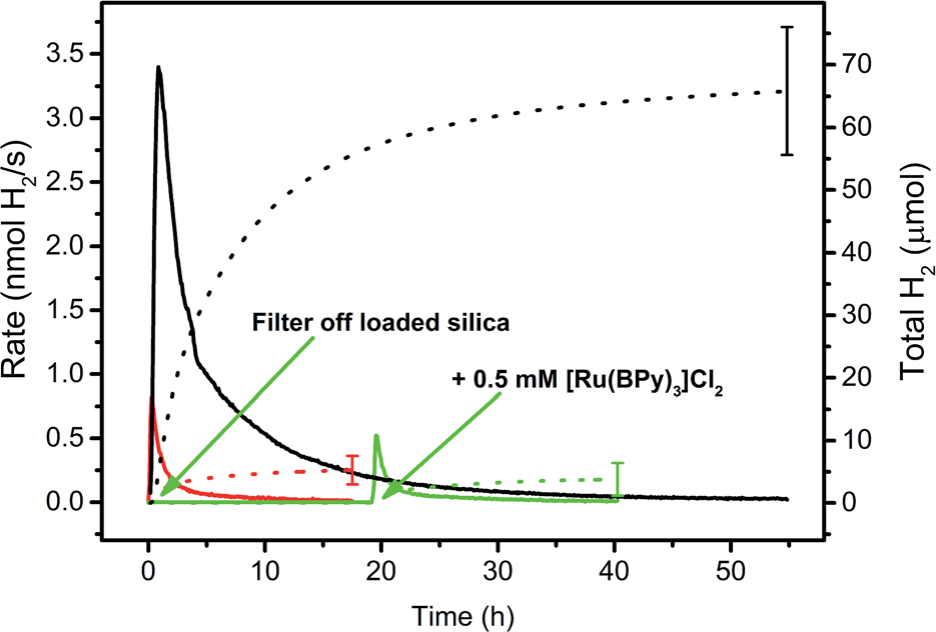
Hydrogen evolution rate courses (solid lines) and total amounts of H_2_ (dotted lines) in 1 M ascorbate buffer (pH 4) with 0.1 M NaOTf. Black: 20 μM **3** and 200 μM **5** adsorbed on hydrophobic fumed silica with 300 μM [C_16_-NMe_3_][OAc] (**7**) as surfactant. Green: same as black, but loaded silica was filtered off and the residual solution irradiated, then 0.5 mM [Ru(bpy)_3_]Cl_2_ was added and irradiation continued (green arrows). Red: 0.5 mM [Ru(bpy)_3_]Cl_2_, no WRC. See Table SI3.†

The suspensions were irradiated with a 453 nm LED and H_2_ evolution continuously measured by automated GC as described earlier.^52^ To make sure that **3** (WRC) or **5** (PS) did not leach from the supports and homogeneous catalysis was observed in fact, the particles were filtered from the suspension after preparation and the residual (colourless) solution irradiated separately (green line in Fig. 2). No H_2_ evolution was observed, supporting retention of WRC and PS on the particles. However, since cobalt polypyridyl complexes are active catalysts already at low concentrations,^52^–^54^ even minute leaching of WRC **3** would result in photocatalysis in the presence of significant amounts of PS **5**. To fully exclude this possibility, we added 500 μM [Ru(bpy)_3_]Cl_2_ to the separated solution and continued irradiation (2^nd^ green arrow in Fig. 2). No increased photocatalytic activity was observed as compared to the blank experiment with no WRC and 500 μM [Ru(bpy)_3_]Cl_2_ only. This conclusively confirmed heterogeneous catalysis on the particles and not from WRC or PS or both eventually released into solution.

### Heterogeneous *vs.* homogeneous photocatalysis

**Fig. 3:**
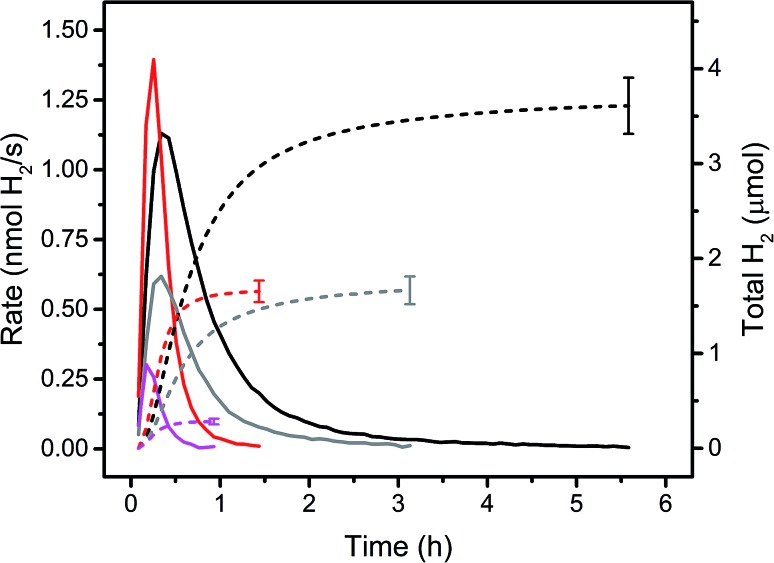
Rates (solid lines) and amounts of H_2_ (dashed lines) for homo- and heterogeneous photocatalytic reactions in 1 M ascorbate buffer (pH 4) with 0.1 M NaOTf. Black: 20 μM PS **5** and 1 μM WRC **3** immobilized on f-SiO_2_-C_18_ with 300 μM **7**. Red: 20 μM [Ru(bpy)_3_]Cl_2_ and 1 μM **1**. Grey: 10 μM PS **5** and 0.5 μM WRC **3** immobilized on f-SiO_2_-C_18_ with 150 μM **7**. Magenta: 10 μM [Ru(bpy)_3_]Cl_2_ and 0.5 μM **1**. Detailed values are shown in Table 1.

Immobilised molecular catalysts give rise to high local concentrations on their respective support in heterogeneous catalysis, whereas in homogeneous catalysis the distribution in solution is even, but concentrations are low. Therefore, it was of interest to compare these two “reversed” situations; activity of PS **5** and WRC **3**, immobilized on f-SiO_2_-C_18_ and [Ru(bpy)_3_]Cl_2_ and **1** in solution, assuming that the alkyl chains in **5** and **3** would not lead to significant activity differences with the latter PS and WRC. Fig. 3 and Table 1 show rates and amounts of H_2_ at different (WRC and PS) concentrations. For the heterogeneous reactions, the amount of f-SiO_2_-C_18_ was reduced in order to keep the loading densities of **5** and **3** constant. At low catalysts concentrations (<20 μM PS and 1 μM WRC), immobilized complexes **5** and **3** exceed the performance of [Ru(bpy)_3_]Cl_2_ and **1** by far (Fig. 3 and Table 1). Apparently, electron transfer from reduced PS to Co^II^ becomes rate limiting in solution at low PS and WRC concentrations. PS^–^ is known to be unstable under aqueous conditions,^61^ therefore at low concentration of **1**, PS decomposition dominates and reaction rates and turnovers drop drastically. In heterogeneous catalysis, the local concentrations on the support silica remain constant and hence catalytic activity of immobilized **5** and **3** is retained even at very low concentrations. This trend is shown in Fig. 3. At 20/1 μM PS/WRC, the immobilized system produces ∼3.5 μmol H_2_, the homogeneous reaction ∼1.5 μmol. At 10/0.5, this ratio becomes larger with ∼1.5 μmol *vs.* ∼0.25 μmol. At lowest concentrations, activity for the homogeneous catalysis is essentially lost, whereas the heterogeneous system remains active. We note a constant TOF in the heterogeneous system at [**3**] ≤ 1 μM (see Table 1), indicating that electron transfer between PS and WRC is not rate limiting, unlike in the homogenous system. At catalyst concentrations ≥100 μM PS and 5 μM WRC, comparable total amounts of H_2_ were observed for both – homo- and heterogeneous catalysis (Table 1). Hence, the stability of immobilized WRC **3** and PS **5** are similar to their homogeneous analogues.

**Table 1:** Summarized results of homogeneous and heterogeneous photocatalytic H_2_ production in 10 mL aqueous 1 M ascorbate buffer (pH 4) with 0.1 M NaOTf. Bold: **3** and **5** immobilized on f-SiO_2_-C_18_, 15 μM surfactant **7** per μM PS **5** and 0.1 M NaOTf. Italic font: homogeneously dissolved [Ru(bpy)_3_]Cl_2_ and **1**. Rate courses and produced hydrogen of 20/1 and 10/0.5 μM PS/WRC are depicted in Fig. 3

[PS]/[WRC] (μM)	Max rate (nmol H_2_/s)	Total H_2_ (μmol)	TON_Co_ (H_2_/Co)
**200/10**	**3.9 ± 0.3**	**79 ± 8**	**790 ± 80**
*200/10*	*12.0 ± 0.75*	*59 ± 4*	*590 ± 40*
**100/5**	**2.85 ± 0.2**	**31.3 ± 2.5**	**626 ± 50**
*100/5*	*11.4 ± 0.7*	*29.5 ± 1.8*	*590 ± 36*
**20/1**	**1.11 ± 0.07**	**3.65 ± 0.35**	**365 ± 35**
*20/1*	*1.38 ± 0.08*	*1.65 ± 0.14*	*165 ± 14*
**10/0.5**	**0.62 ± 0.04**	**1.7 ± 0.2**	**340 ± 40**
*10/0.5*	*0.30 ± 0.02*	*0.28 ± 0.03*	*56 ± 6*
**2/0.1**	**0.09 ± 0.009**	**0.20 ± 0.04**	**200 ± 40**
*2/0.1*	*<0.01*	—	—

However, homogeneous catalysis exhibited 3–4 fold higher H_2_ rates (Table 1). Presumably, two effects are responsible; (i) light is more uniformly absorbed in homogeneous solution and therefore more PS activated and (ii) reductive quenching of excited PS (Ru*) is significantly slower in heterogeneous catalysis as ascorbate diffusion to silica is limiting. Consequently, in this concentration range, distinctly higher H_2_ evolution rates were achieved with [Ru(bpy)_3_]Cl_2_ and **1**. Apart from these limitations, immobilization does not adversely affect the catalyst performance since TONs for both systems are about identical (Table 1 and Fig. 3).

### Concentration dependencies

**Fig. 4:**
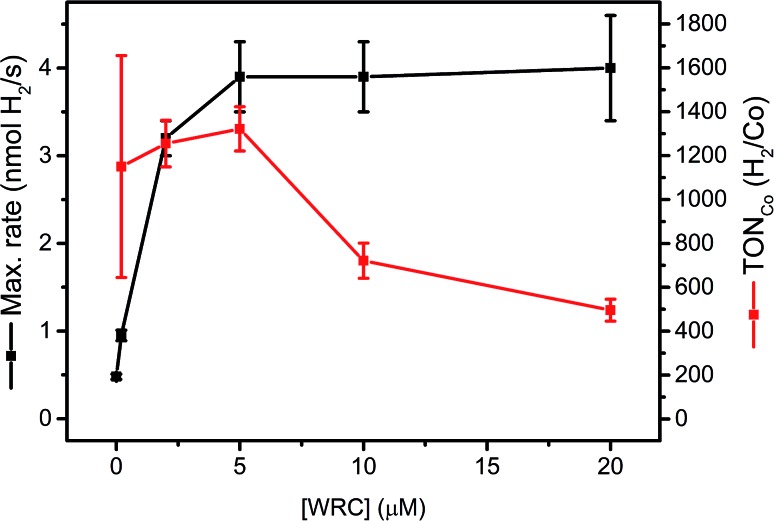
[WRC] dependency study with 200 μM PS **5** and varying amounts of WRC **3** (0, 0.2, 2, 5, 10 and 20 μM) adsorbed on f-SiO_2_-C_18_ in 1 M ascorbate buffer, 0.1 M NaOTf and 300 μM [C_16_-NMe_3_][OAc] (**7**) as surfactant. The amount of H_2_ from the blank experiment (no WRC) was subtracted at each concentration and the corresponding TONs in Co (H_2_/Co) calculated (Table SI6†).

Complex **1** is a highly active WRC which achieves TON_Co_ as high as 30 000 H_2_/Co, when back electron transfer is inhibited by the regeneration of DHA with tris-(2-carboxyethyl) phosphine (TCEP).^52^ Catalytic performance as a function of the WRC concentration provides insights in process limitations. We therefore varied systematically the concentration of WRC **3**, while keeping PS **5** and all other parameters constant. If mobility's of **3** and **5** on f-SiO_2_-C_18_ were low, WRC “dilution” will entail a linear decrease in rates with concentrations. Two distinct domains can be seen in Fig. 4: a constant H_2_ evolution rate, accompanied by a decrease in TON_Co_ between 5 and 20 μM WRC and a linear decrease in rate, accompanied by constant TON_Co_ below [WRC] = 5 μM.

In the first domain, as for homogenous catalysis, we assume the PS (**5**) cycle becomes rate limiting (number of photons, diffusion of ascorbate, quench yield). Although [Ru(bpy)_3_]^2+^ is known to undergo rapid electron self-exchange reactions (PS + PS^–^ ↔ PS^–^ + PS) at an estimated rate of ∼10^8^ M^–1^ s^–1^,^62^ the mean Ru–Ru distance under these conditions (∼3–4 nm at 0.15 μmol m^–2^) is too large for efficient electron hopping to the WRC, if catalysts were not mobile. We thus conclude that **3** and **5** remain mobile on hydrophobic silica which enables rapid dynamic reorganization. The surface can be considered as a two dimensional liquid in interfacial contact with an aqueous solution. König and co-workers observed a similar mobility for complexes embedded into membranes.^45^ The similar amounts of H_2_ obtained in the first domain (Table SI6†) also indicate PS degradation. A recycling experiment at high [WRC] further corroborated this hypothesis: replacing the catalysis medium by fresh ascorbic acid buffer solution after 20 h and 30 h of irradiation gave significantly increased reaction rates, whereas the total amount of evolved H_2_ was similar compared to other reactions with high [WRC] (Fig. SI9 and Table SI6†). Consequently, catalysis is slowed down by DHA accumulation, but PS bleaching limits the stability.

In the second domain, below 5 μM [WRC], we assume that the WRC cycle limits the rate. In contrast to the homogeneous reaction, TON_Co_ did not increase in this domain, but remained constant (1300 H_2_/Co). As the stability of **3** is expected to be similar to **1** (>30 000 H_2_/Co (ref. 52)) we hypothesize mobility limitation in the second domain: since reduced PS is unstable under aqueous conditions,^61^ turnovers are limited if electron transfer to **3** (**5^–^** + **3** → **5** + **3^–^**) becomes too slow at low [WRC]. Thus only reduced PS within a maximal distance of a WRC molecule can deliver its reduction equivalents, whereas those that are too far away will decompose.

Variation of the surface concentration of **3** and **5** (5 resp. 100 μM) on varying amounts of f-SiO_2_-C_18_ (0.02–0.27 molecules per nm^2^) gave a decrease of catalysis rate at low loading, whereas constant rates were observed above (Table SI4†).

**Fig. 5:**
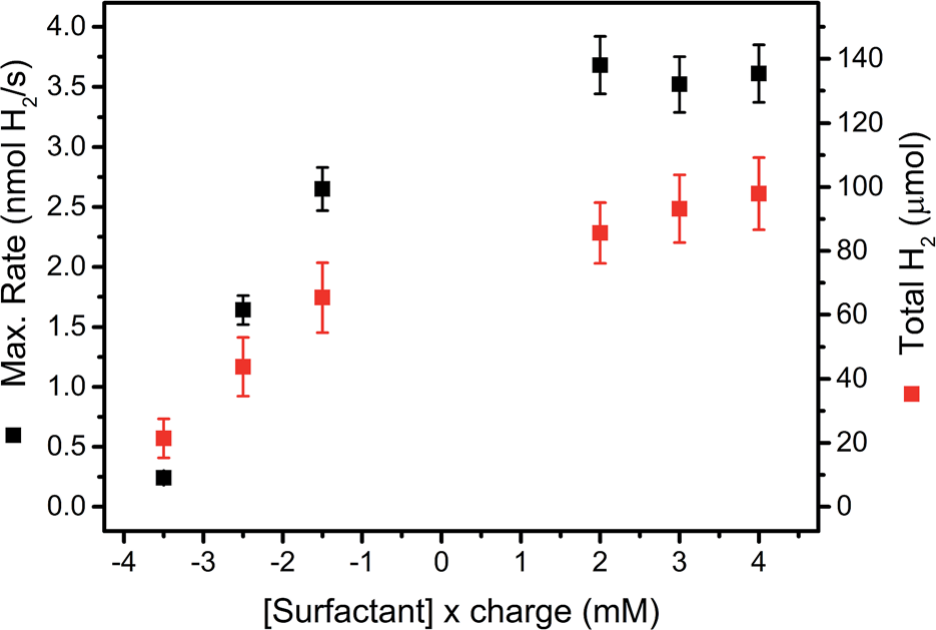
Maximal H_2_ evolution rates (black squares, left scale) and total amounts of evolved H_2_ (red squares, right scale) in 1 M ascorbate buffer (pH 4) with 0.1 M NaOTf and 200 μM PS **5** and 20 μM WRC **3** immobilized on f-SiO_2_-C_18_ at different surfactant concentrations (mM) multiplied by their charge (negative: Na[C_12_-PhSO_3_], **6**; positive: [C_16_-NMe_3_][OAc], **7**). Results are summarized in Table SI5† and the structures of **6** and **7** depicted in Scheme SI1.†

Maximal H_2_ evolution rates and total amounts of evolved H_2_ at different concentrations of surfactants **6** (anionic) and **7** (cationic) are shown in Fig. 5. Both, maximal H_2_ evolution rates and total H_2_ amounts increased distinctly with decreasing concentrations of anionic surfactant **6** and otherwise identical conditions. Cationic surfactant **7**, on the other hand, gave higher catalytic activity and stability in the concentration range accessible for catalysis. Experiments using surfactants **6** and **7** in a homogenous system gave no significant effect on the rate of photocatalysis (Table SI2†). Electrostatic surface repulsion is thus a likely explanation in the immobilized system, as negatively charged ascorbate is attracted by a support surface covered with cationic **7**, whereas diffusion of ascorbate onto silica is impeded by negatively charged **6**. Thus reductive quenching of excited PS (Ru*) becomes slower which entails a decreased reaction rate. In parallel, electron back transfer to the continuously formed, neutral DHA is reduced which results in more H_2_ with the cationic surfactant **7**. Cationic surfactants are therefore superior to anionic counterparts in heterogeneous photocatalytic H_2_ production.

### Different silica supports

**Fig. 6:**
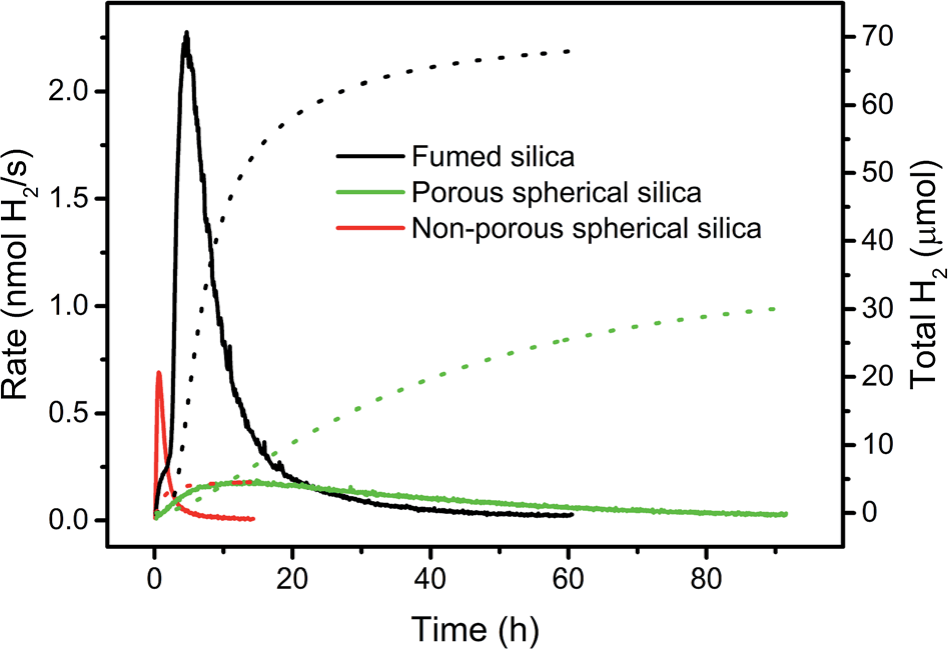
Comparison of rates (solid lines, left scale) and H_2_ amounts (dotted lines, right scale) with WRC **3** and PS **5** immobilized on different types of hydrophobic silica in 1 M ascorbate buffer (pH = 4, 0.1 M NaOTf). Black: 200 μM PS **5** and 40 μM WRC **3** on f-SiO_2_-C_18_ with 2.5 mM **6**. Red: 4.8 μM **5** and 1 μM **3** on non-porous spherical silica with 100 μM **6**. Green: 200 μM **5** and 40 μM **3** on porous silica with 2.5 mM **6**.

A high BET surface area, accessible for light and reaction components is crucial for catalytic activity. To support this hypothesis, we compared the performance of WRC **3** and PS **5** immobilized on three silica-based supports with different BET surface areas and accessibilities, but with similar particle diameters. Commercially available C_18_-silica (*d* = 40–70 μm) as used in reverse phase column chromatography is highly porous (pore size: 7 nm; pore volume: 0.7–0.9 cm^3^ g^–1^) and has a very large BET surface area (480 m^2^ g^–1^). Catalysts adsorbed in the pores might show altered kinetics (local accumulation of oxidised ascorbate, inhibition), and catalytic activity should therefore be low. Calcination of porous silica (*d* = 10 μm) at 1100 °C followed by C_18_-grafting, gives spherical, non-porous hydrophobic silica (*d* = 7–9 μm, Scheme SI5†) with a good accessibility but low BET surface area (*ca.* 0.5 m^2^ g^–1^, see Experimental part). These two silica based supports were compared to f-SiO_2_-C_18_, which is non-porous but consists of small silica chains and branches, aggregated to larger particles (*d* = 0.2–50 μm, Fig. SI3†) with a high BET surface area (200 m^2^ g^–1^). Thus, fumed silica combines both advantages of porous and non-porous silica; a high BET surface area and good accessibility. As expected, porous silica showed an even distribution of PS through the whole particle – similarly to f-SiO_2_-C_18_ aggregates – whereas for non-porous silica PS was only observed on the particle surface (Fig. SI5–SI8†). H_2_ evolution rates and total amounts of H_2_ with **3** and **5** immobilized in these three supports are depicted in Fig. 6. Under comparable conditions, catalysts grafted to fumed silica exhibited a 10 times higher activity and the double amount of H_2_ in half the time as compared to porous silica (Fig. 6). Not all sites in porous silica are equally accessible to ascorbate and protons, and high local concentrations of DHA might inhibit catalysis. Consequently, only a fraction of the immobilized PS contributes to catalysis and lower rates are the result. Accessibility is thus an important factor, and a high BET surface area alone is not sufficient. A direct comparison to non-porous silica is difficult. Only small amounts of catalysts (30–40 times less) can be grafted as non-porous silica has only a very low BET surface area (*ca.* 0.5 m^2^ g^–1^). Yet, **3** and **5** exhibited a 2–3 times higher maximal H_2_ evolution rate on non-porous compared to porous silica albeit H_2_ evolution ceased quickly due to low amounts of catalysts (Fig. 6). These results corroborate the importance of a high BET surface area and non-porosity as an essential base for efficient photocatalysis. Large quantities of well accessible catalysts can be adsorbed to maintain H_2_ formation over a long period, and high reaction rates are achieved through good accessibilities. Fumed silica is an excellent and cheap support for heterogeneous catalysis with immobilized molecular complexes.

### Mechanisms

**Scheme 4:**
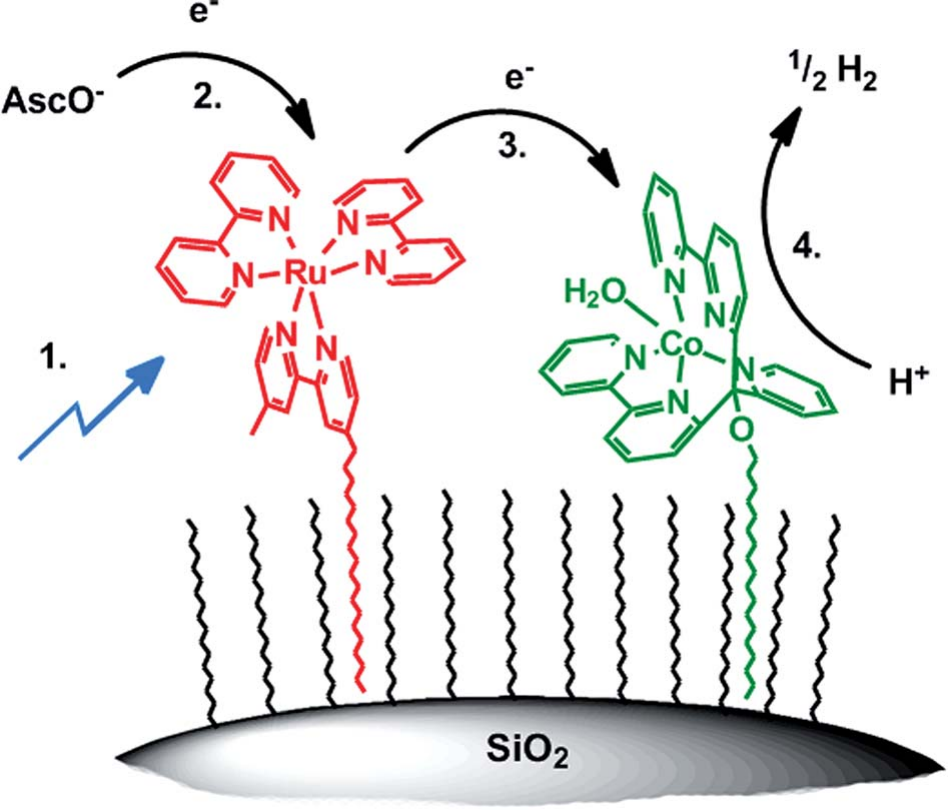
Proposed reaction mechanism of photocatalytic H_2_ production on hydrophobic silica: (1) photoexcitation of **5** → 5*; (2) reductive quenching of 5* → **5^–^**; (3) electron transfer **5^–^** → **3**; (4) H^+^ reduction by **3^–^**.

For obtaining a closer insight into the mechanism of H_2_ formation and electron transfer in particular, we performed transient absorption spectroscopy of unsupported catalysts [Ru(bpy)_3_]Cl_2_ and **1**. There are two possible pathways; excited PS* is reductively quenched by H_asc_^–^ followed by electron transfer to **1** and subsequent proton reduction (Scheme 4; 1.–4.) or oxidative quenching of Ru* by **1** followed by reduction of oxidized [Ru(bpy)_3_]^2+^ (Ru^III^) with H_asc_^–^. Although less likely, the second pathway cannot be excluded “*a priori*” since on the particle, **3** and **5** are in intimate vicinity. Transient absorption spectroscopy in water with [CoBr(appy)]Br (**1**) and [Ru(bpy)_3_]Cl_2_ did, however, not show any detectable formation of Ru^III^ or Co^I^ species, but non-productive quenching, most likely by energy transfer from PS* to **1** with *k*_q_ = 2.2 ± 0.1 × 10^8^ M^–1^ s^–1^ (Fig. SI10†). In contrast, excitation of [Ru(bpy)_3_]^2+^ in ascorbate buffer resulted in the immediate formation of reduced PS, with *k*_q_ = 2.7 ± 0.3 × 10^7^ M^–1^ s^–1^ (Fig. SI11 and SI12†), consistent with our results on ascorbate diffusion (see Section Surfactant dependency) and results from literature.^48^,^63^–^65^ According to previous kinetic studies with other PSs and cobalt based WRCs, a fast electron transfer from reduced PS to WRC (**PS^–^** → **1**) was expected.^56^,^66^–^68^ The electron transfer rate of photogenerated **PS^–^** to **1** is indeed close to diffusion control with *k*_ET_ = 1.6 ± 0.1 × 10^9^ M^–1^ s^–1^ (Fig. SI13 and SI14†). This rate coincides well with values reported previously for other poly-pyridyl based Co WRCs by Chang or Scandola and co-workers.^64^,^65^ We tentatively propose a similar mechanism for the immobilized systems: excitation of **5** → 5*, followed by reductive quenching by H_asc_^–^ from bulk solution and electron transfer to **3**. The model rationalizes also the increased catalytic performance of cationic (**7**) over anionic surfactants (**6**). According to our detailed and recent study with a similar tetra-pyridyl based Co WRC, we propose the final formation of H_2_ to occur by protonation of Co^I^ → Co^III^–H, reduction of Co^III^–H → Co^II^–H and subsequent protonation and H_2_ release.^69^

## Conclusion

A convenient method to immobilize molecular PSs and WRCs on hydrophobic supports by non-covalent interactions is presented. This photocatalytic architecture can be applied to various molecular PSs and WRCs since derivatizations with long alkyl chains are synthetically versatile, and no active functionalities are present, potentially interfering with the basic catalyst framework. The approach allows moving from homogeneous to heterogeneous catalysis, while mechanistic insights can still be obtained from studies in solution. We exemplify this concept with WRC **3** and PS **5**. No leaching was observed, in agreement with strong hydrophobic interactions. At low concentrations, catalytic activity was still observed while the homogenous systems ceased to produce H_2_. At high concentrations, the homogenous system exceeds the immobilised one in terms of rate, but stability is retained. A proper choice of surfactants improved performance without altering the basic catalytic system. A careful analysis of the concentration dependency showed that immobilized catalysts remained mobile on their supports, and that PS stability limits turnover of catalysis, in line with results from recycling experiments. In summary, highly active molecular catalysts were immobilized by a straightforward and flexible method on cheap and robust supports, which is an important step towards heterogenization and thus physical separation of HER and potentially OER in a molecular water splitting architecture.

## Experimental

### Syntheses

Poly-pyridine ligand **2** (appy), [Ru(bpy)_2_Cl_2_] and [Ru(bpy)_3_]Cl_2_ were prepared according to reported procedures.^54^,^70^,^71^

#### [Co(C_18_H_37_-appy)(H_2_O)](ClO_4_)_2_ (**3**)

Ligand **2** (appy, 200 mg, 0.48 mmol, 1 eq.) and NaH (60% in mineral oil; 55.1 mg, 1.44 mmol, 3 eq.) were stirred in dry DMF (20 mL) for 45 min at rt. Iodo-octadecane (913 mg, 2.4 mmol, 5 eq.) was added and stirring continued for 15 h at rt. CH_2_Cl_2_ and aq. NaOH (*ca.* 2 M) were added and the phases separated. The aqueous phase was washed three times with CH_2_Cl_2_, the combined organic phases dried over MgSO_4_, filtered and concentrated. The resulting yellowish oil was dissolved in MeOH/Et_2_O (3 : 1, 20 mL) and Co(ClO_4_)_2_ hexahydrate (175.6 mg, 0.48 mmol, 1 eq.) added. The resulting brown solution was stirred for 3 h at rt followed by precipitation with excess Et_2_O. The solid was filtered off, washed 3× with Et_2_O and dried *in vacuo* to give **3** as light brown solid (358 mg, 0.38 mmol, 79%).ESI-MS (MeOH): *m*/*z* = 364 [M – 2 ClO_4_]^2+^ (100%), [M – ClO_4_]^+^ (50%), elemental analysis: calcd. for [Co(C_18_H_37_-appy)(H_2_O)](ClO_4_)_2_ (%): C, 55.88; H, 6.07; N, 7.40. Found: C, 56.05; H, 5.98; N, 7.29; UV/vis absorption (MeOH): *λ*_max_ = 248 nm (*ε* = 1800 M^–1^ cm^–1^); 299 nm (*ε* = 2400 M^–1^ cm^–1^); 455 nm (shoulder, *ε* = 60 M^–1^ cm^–1^).

#### 4-Methyl-4′-nonadecyl-2,2′-bipyridine (**4**)

4,4′-Dimethyl-2,2′-bipyridyl (0.1 g, 0.54 mmol, 1 eq.) was suspended in dry THF (3 mL) and cooled to –45 °C in an MeOH/dry ice bath. LDA (2 M in THF, 0.54 mL, 1.1 mmol, 2 eq.) was slowly added over 30 minutes and the mixture stirred for 2 h at –45 °C. Afterwards, a solution of 1-bromooctadecane (271 mg, 0.65 mmol, 1.2 eq.) in dry THF (1 mL) was added drop wise to the reddish/brown solution, and the reaction mixture allowed to warm up to room temperature and further stirred for 15 h. The reaction mixture was quenched with MeOH (3 mL) and stirred for another 30 min at room temperature followed by concentration. The crude product was purified by flash chromatography (silica gel, EtOAc) to give **4** as colourless solid (209 mg, 0.48 mmol, 88%). ^1^H NMR (300 MHz, CDCl_3_, ppm): *δ* = 8.55 (dd, *J* = 5.1 and 3.0 Hz, 2H), 8.23 (unresolved d, 2H), 7.14 (dd, *J* = 5.1 and 1.5 Hz, 2H), 2.70 (t, *J* = 8.1 Hz, 2H), 2.45 (s, 3H), 1.72–1.64 (m, 2H), 1.26 (br s, 32H), 0.89 (unresolved t, 3H). ^13^C[^1^H] NMR (75 MHz, CDCl_3_, ppm): *δ* = 152.9, 148.9, 148.1, 128.4, 127.0, 125.6, 124.6, 123.9, 122.0, 121.3, 43.8, 35.8, 35.6, 31.9, 31.1, 30.5, 29.7, 29.54, 29.49, 29.44, 29.38, 29.2, 23.9, 22.7, 21.2, 14.1. ESI-MS (MeOH): *m*/*z* = 437.7 [M + H]^+^ (100%). Elemental analysis: calcd for C_30_H_48_N_2_ (%): C, 82.51; H, 11.08; N, 6.41. Found: C, 82.55; H, 11.04; N, 6.38.

#### [Ru(bpy)_2_(**4**)](PF_6_)_2_ (**5**)

[Ru(bpy)_2_Cl_2_]·2H_2_O (300 mg, 0.62 mmol, 1 eq.) and **4** (543 mg, 1.24 mmol, 2 eq.) were suspended in H_2_O/EtOH (1 : 1, 130 mL) and heated up to 100 °C for 24 h. The hot reaction mixture was filtered through celite, cooled down to rt and concentrated. The residual solid was washed 3× with 10 mL of hexane and dissolved in water. A threefold excess of NH_4_PF_6_ as concentrated aqueous solution was added dropwise to precipitate the complex as PF_6_–salt. Filtration and washing with water followed by drying gave **5** as red solid (498 mg, 0.44 mmol, 71%). UV/vis absorption (H_2_O): *λ*_max_ = 453 nm (*ε* = 13 300 M^–1^ cm^–1^). Luminescence (H_2_O): *λ*_em_ = 623 nm (*Φ*_P,abs_ = 0.07; *τ* = 789 ns). ^1^H-NMR (300 MHz, CDCl_3_, ppm) *δ* = 9.04–8.99 (m, 4H), 8.56 (s, 1H), 8.49 (s, 1H), 8.17–8.10 (m, 4H), 7.81–7.73 (m, overlapping signals, 4H), 7.63–7.48 (m, overlapping signals, 6H), 7.36–7.29 (m, overlapping signals, 2H), 2.86 (t, *J* = 7.8 Hz, 2H), 2.64 (s, 3H), 1.73 (unresolved quint., 2H), 1.26 (br s, 32H), 0.88 (t, *J* = 6.6 Hz, 3H). ESI-MS (MeOH): *m*/*z* = 425.4 [M]^2+^ (100%), elemental analysis: calcd for C_50_H_64_N_6_P_2_F_6_Ru (%): C, 52.67; H, 5.66; N, 7.37. Found: C, 52.42; H, 5.60; N, 7.31.

#### [C_16_-NMe_3_][OAc] (**7**)

0.1 M aqueous stock solution of **7** was obtained by stirring *N*,*N*,*N*-trimethylhexadecyl ammonium hydroxide ([C_16_-NMe_3_][OH]; 25% in MeOH, 1.470 mL, 1 mmol) and acetic acid (58 μL, 1 mmol) in 7 mL water for 10 min at rt. The MeOH was evaporated and the solution diluted with water up to 10 mL.

### Preparation of different supports

#### Spherical porous silica

Commercially available hydrophobic silica (45–70 μm, 480 m^2^ g^–1^) was directly used without further modification.

#### Spherical non-porous silica

Porous spherical silica (20 g, *d* = 10 μm, 340 m^2^ g^–1^) was glowed in an Alox crucible at 1100 °C for 12 h. The resulting solid was suspended in 10% aqueous HNO_3_ (110 mL) and refluxed for 5 h followed by filtration and washing with water until the wash solution had pH > 5. Surface measurement by N_2_ adsorption (BET) gave a surface of 0.459 m^2^ g^–1^. The product was dried *in vacuo* for 5 h and then stirred overnight (15 h) in a solution of trichlorooctadecylsilane (5 mL) in DCM (50 mL). The slurry was filtered, the resulting solid washed with DCM and MeOH and finally concentrated and dried *in vacuo*. FM measurements of loaded particles showed a particle size of ∼7–9 μm (Scheme SI5†).

#### Fumed silica

Hydrophilic fumed silica (6 g, 200–300 nm, 200 m^2^ g^–1^) was stirred overnight (15 h) in a solution of trichlorooctadecylsilane (4 mL) in DCM (180 mL). The suspension was centrifuged, the particles washed with DCM and MeOH, concentrated and dried *in vacuo*.

### Adsorption of catalysts

Standard procedure to load catalysts on hydrophobic silica: Hydrophobic fumed silica (200 m^2^ g^–1^; 447 mg = 89.4 m^2^ BET surface area) and the corresponding amounts of WRC **3** and PS **5** (totally 13 μmol, added from freshly prepared methanolic stock solutions, giving a loading of 0.15 μmol per m^2^ silica surface) were suspended in MeOH (15 mL) and stirred for 30 min. Then 0.1 M aqueous surfactant solution (300 μL **6** or **7**) was added and the mixture stirred for an additional 10 min followed by addition of aqueous electrolyte solution (0.1 M NaOTf, 15 mL). The mixture was warmed up to 50 °C in a water bath and the MeOH evaporated with a N_2_ flow. The resulting aqueous suspension was sonicated for 5 min and centrifuged. The solution was decanted and analyzed by HPLC to ensure full adsorption of catalysts (filtration through 0.22 μM syringe filter prior to measurement). The resulting solid was dried at 50 °C with a N_2_ flow to give an orange powder.

### Catalysis

Standard preparation of catalysis suspensions/solutions: In a graduated cylinder sodium ascorbate (990.5 mg, 5 mmol) and ascorbic acid (880.5 mg, 5 mmol) were dissolved in water and diluted to 9 mL.

#### Heterogeneous catalysis

Weighted amounts of silica particles with adsorbed PS **5** and WRC **3** were placed in the reaction vessel and the ascorbate buffer solution added. Then the corresponding amount of surfactant was added (**6** or **7**, as 0.1 M aqueous solution) and the particles properly suspended by stirring and sonication. Finally NaOTf (171 mg, 1 mmol) was added as solid and the suspension diluted up to 10 mL. The resulting reaction mixture was analyzed by HPLC (filtered through 0.22 μm syringe filter) to exclude leaching of catalysts.

#### Homogeneous catalysis

The ascorbate buffer solution was transferred into the reaction vessel and corresponding amount of [Ru(bpy)_3_]Cl_2_ and WRC **1** added. Finally NaOTf (171 mg, 1 mmol) was added as solid and the solution diluted up to 10 mL.

The reaction mixtures were connected to an automated sampling system linked to a GC-TCD, degassed with Ar followed by illumination with 453 nm LED (85 ± 2 mW; photon flux: 3 × 10^–7^ mol s^–1^). H_2_ evolution was measured as described in a previous publication.^52^

## Supplementary Material

Supplementary information
