# Supplementary material for: Photocatalytic proton reduction with ruthenium and cobalt complexes immobilized on fumed reversed-phase silica
Source: Chem Sci. 2015 Oct 8;7(1):436–45. doi: 10.1039/c5sc02124c (PMC5952309; doi:10.1039/c5sc02124c)
Supplement: Supplementary file 1 [file SC-007-C5SC02124C-s001.pdf]

Electronic Supplementary Material (ESI) for Chemical Science.
This journal is © The Royal Society of Chemistry 2015

Supporting Information for

Photocatalytic Proton Reduction with Ruthenium and Cobalt Complexes Immobilized on Fumed Reversed-Phase Silica

Cyril Bachmann, Benjamin Probst, Miriam Oberholzer, Thomas Fox and Roger Alberto

Table of Contents

1. General 2
2. ^{13}C solid and solution NMR spectra of PS 5 4
3. Structures of used surfactants 5
4. DLS measurements 5
5. Fluorescence microscopy: 6
6. Additional tables and figures 9
7. Transient absorption measurements 12

1. General

All dry and air free **reactions** were performed on a standard nitrogen/vacuum line in glassware dried at 140°C at least overnight. THF was dried over Na/Benzophenone and dry DMF purchased from Acros. All other solvents were of analytical grade and used without further purification. Water for catalytic reaction was doubly distilled before use and extractions and column chromatographies were done with normally distilled technical solvents.
Chemicals: Trichlorooctadecyl silane, sodium hydride (60 % dispersion), 1-iodooctadecane, 1-bromooctadecane, nitric acid, hydrophilic fumed silica, porous hydrophobic silica (45-70 µm, pore size: 7 nm, pore volume: 0.7-0.9 cm^3/g), sodium 4-dodecylbenzenesulfonate (Na[C_{12}-$PhSO_3$]), sodium ascorbate, ammonium hexafluorophosphate and lithium diisopropylamine (LDA, 2 M in THF) were purchased from sigma aldrich, porous hydrophilic silica (d = 10 µm) from Marchery Nagel, ascorbic acid from Acros, acetic acid and ethanol from Merck and 4,4'-dimethyl-2,2'-bipyridyl, ,N,N,N-trimethylhexadecyl ammonium hydroxide solution (25 % in MeOH) and sodium trifluoromethanesulfonate (NaOTf) from TCI.
All products were directly used without further purification and syntheses were monitored by HPLC measurements.
^{1}H- and ^{13}C-NMR measurements (200 MHz or 300 MHz) were performed on a Varian Mercury or a Varian Gemini-2000 spectrometer. Chemical shifts are reported relative to the residual solvent peaks. Solid and solution NMR of PS **5** shown below were run on a Bruker AV3-500 MHz NMR spectrometer (500.25 MHz ^{1}H- and 125.78 MHz ^{13}C- frequency). The solid state ^{13}C measurements have been carried out under Hartman-Hahn condition and magic angle spinning (^{13}C-CPMAS) with high power ^{1}H-decoupling.
Elemental analyses were performed on a Leco CHNS-932 elemental analyzer.
ESI-MS were recorded on a Brucker HCT ultra mass spectrometer.
UV/VIS absorption spectra were measured on a Varian Cary50Scan UV-Visible Spectrophotometer.
Luminescence spectra were recorded on a Perkin Elmer LS 50B.
HPLC measurements were performed on a VWR Hitachi Elite LaChrome with a C_{18}-Phenomenex Core Shell column and H_2O/MeOH as eluent (gradient starting with 10 % MeOH, 0.1 % TFA in H_2O to pure MeOH).
Lifetime and transient absorption measurements were recorded on an Edinburgh LP920 Laser Flash Photolysis transient absorption spectrometer using a flash lamp pumped Q-switched Nd:Yag laser (355 nm) as excitation source. Samples were degassed by bubbling with N_2 prior to measurements. The laser power was adjusted to ca 1.2mJ/shot in order to avoid double excitation of Ru PS as observed by Brettel et al.[1] Relevant components of a singular value decomposition (SVD) analysis were exponentially fitted to determine the corresponding reaction rates.
Transmission electron microscopy (TEM) was done on a FEI Tecnai G2 Spirit operated at 120 kV. One drop of a dilute aqueous silica suspension was placed onto a formvar-coated copper grid, left for ca 30 s followed by removing residual liquid with a tissue and drying the sample overnight at rt.
Fluorescence microscopy was performed on a Leica SP5 Confocal laser scanning microscope (Objective: 63 x 1.4; oil immersion).
Dynamic light scattering (DLS) measurements were performed on a DynaPro Titan machine equipped with a temperature controlled microsampler from Wyatt Technology Corporation. Data were analyzed with the Dynamics V6 software.

The **surface area** was determined by the Brunauer−Emmett−Teller **(BET)** method on a Quadrasorb SA 6 in N_2 adsorption mode. Samples (ca. 300 mg) were degassed at 423 K for 24 h in vacuo prior to nitrogen adsorption measurements with a Quantachrome FLO VAC degasser.

2. ^{13}C solid and solution NMR spectra of PS 5

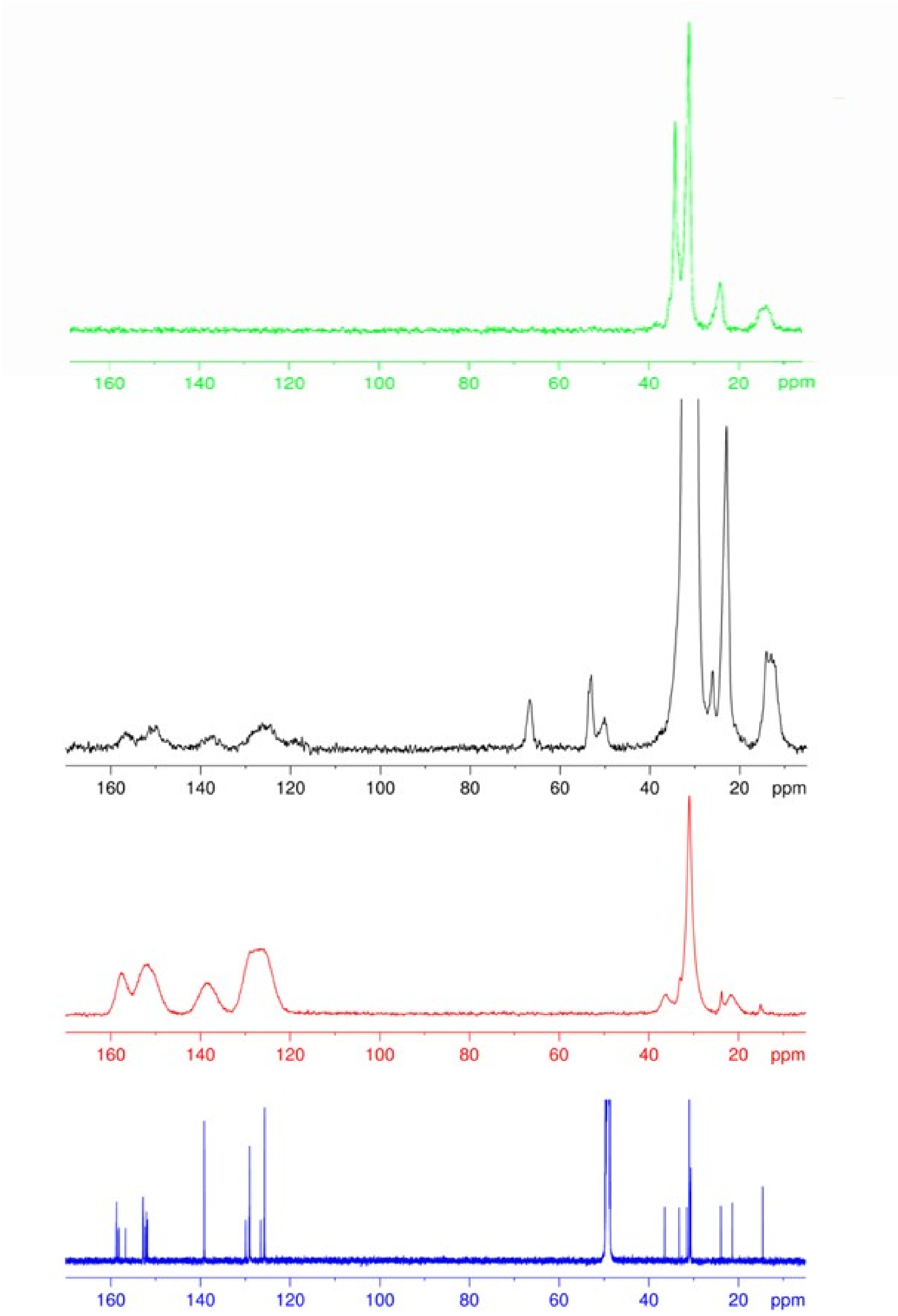

Figure SI1: **Green:** ^{13}C-CPMAS solid state NMR of f-SiO_2-C_{18} without adsorbants. **Black:** ^{13}C CPMAS solid state NMR of PS **5** adsorbed on f-SiO_2-$C_{18.}$ The additional peaks between 40 and 80 ppm are assigned to surfactant **7**, which was adsorbed during immobilization of PS and WRC. **Red:** ^{13}C-CPMAS solid state NMR of pure PS **5**. **Blue:** ^{13}C-NMR of PS **5** in MeOD.

3. Structures of used surfactants

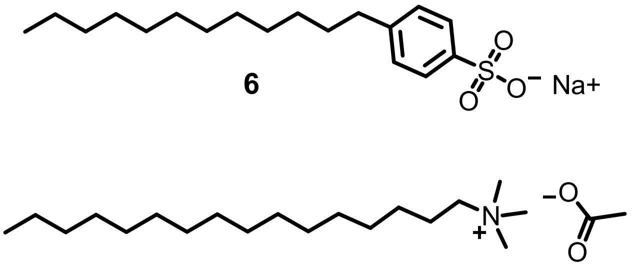

6

7

Scheme SI1: Structures of the used surfactants.

4. DLS measurements

Stock suspensions for DLS measurements were prepared at the same concentrations as catalytic suspensions (7-8 mg/mL) and diluted 1:1000 with water prior to measurements.

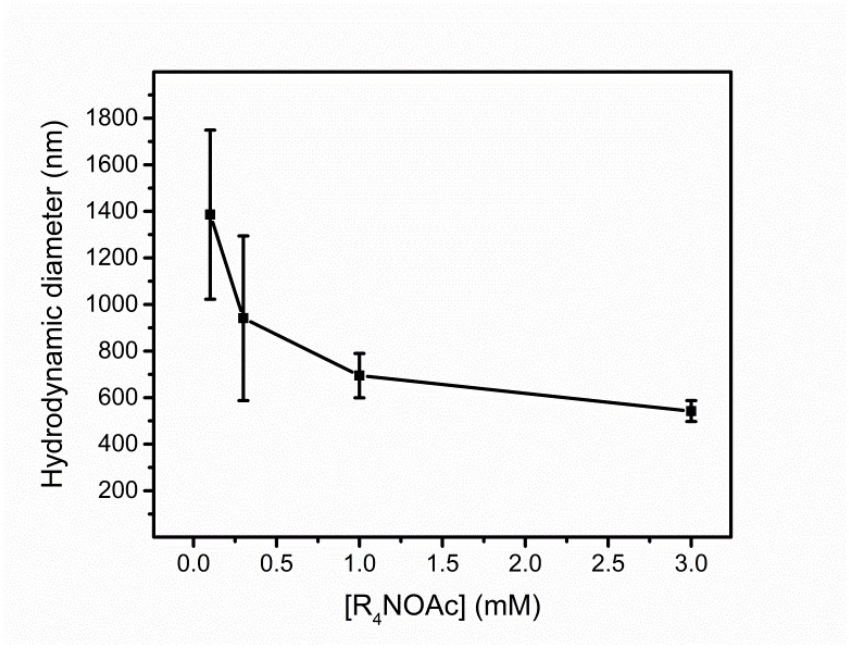

Figure SI2: Mean hydrodynamic diameter of hydrophobic fumed silica loaded with PS **5** and WRC **3** (ratio 10:1, 0.15 µmol per m^2) suspended in water with different amounts of [C_{16}-NMe_3][OAc] (**7**, 0.1, 0.3, 1 and 3 mM). For DLS measurements the solution were diluted 1:1000 with water. Suspending particles with 10 mM or more surfactant lead to release of catalysts from the particles and without **7** loaded silica could not be properly suspended.

Table SI1: Mean hydrodynamic diameters and distributions of hydrophobic fumed silica under different conditions determined by DLS measurements. Solutions were diluted 1:1000 with water prior to measurement.

Conditions	Mean diameter (nm)
Hydrophobic fumed silica, no catalyst adsorbed, 3 mM aqueous [C_{16}-NMe_3][OAc] (**7**).	280 ± 13
Hydrophobic fumed silica, **3** and **5** adsorbed, 3 mM aqueous [C_{16}-NMe_3][OAc] (**7**).	540 ± 45
Hydrophobic fumed silica, **3** and **5** adsorbed, 3 mM aqueous [C_{16}-NMe_3][OAc] (**7**) and 0.1 M NaOTf electrolyte.	2500 ± 2000
Hydrophobic fumed silica, **3** and **5** adsorbed, 3 mM aqueous [C_{16}-NMe_3][OAc] (**7**) and 1 M sodium ascorbate/ascorbic acid buffer (1:1).	1050 ± 170

5. Fluorescence microscopy:

Suspensions for FM measurements were prepared the same way as for catalysis but without ascorbate buffer. One drop (ca 10 µL) was placed between two glass plates separated by a sticking mask (d = 100 µm) with a measurement window (r = 3 mm).

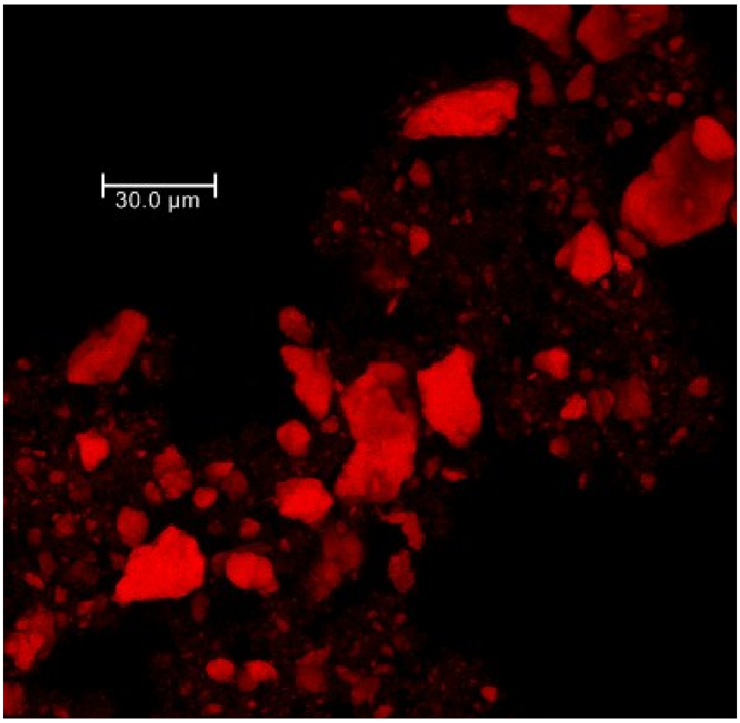

Figure SI3: Representative 3D-projection of fumed silica loaded with PS **5** in water (7-8 mg/mL) with 3 mM cationic surfactant (**7**) and 0.1 M NaOTf. The projection was made of 24 pictures at different depths with a distance of 3 µm between each layer.

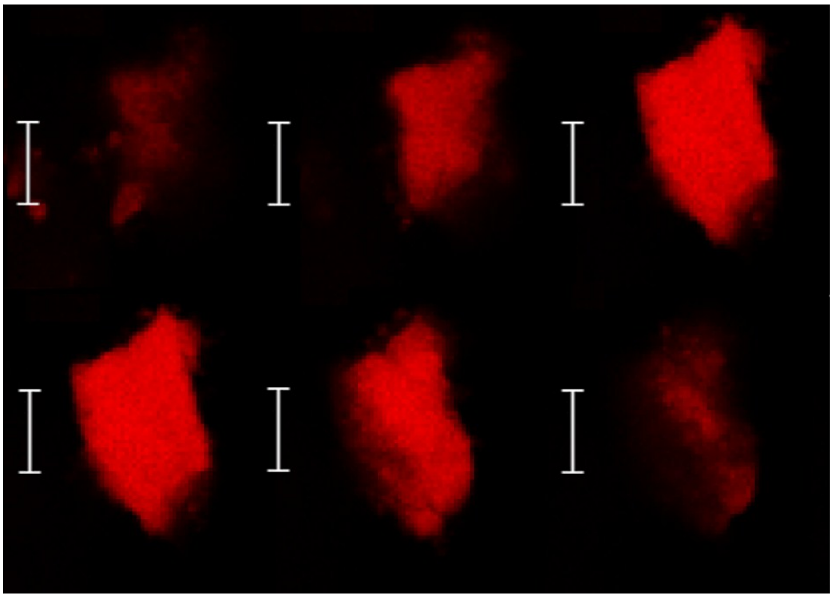

Figure SI4: Representative fluorescence micrograph of a selected aggregate of loaded fumed silica at different depths (z-direction). The conditions are the same as in Figure SI3, the length of the scale bar is 10 µm and the distance between each picture 3 µm.

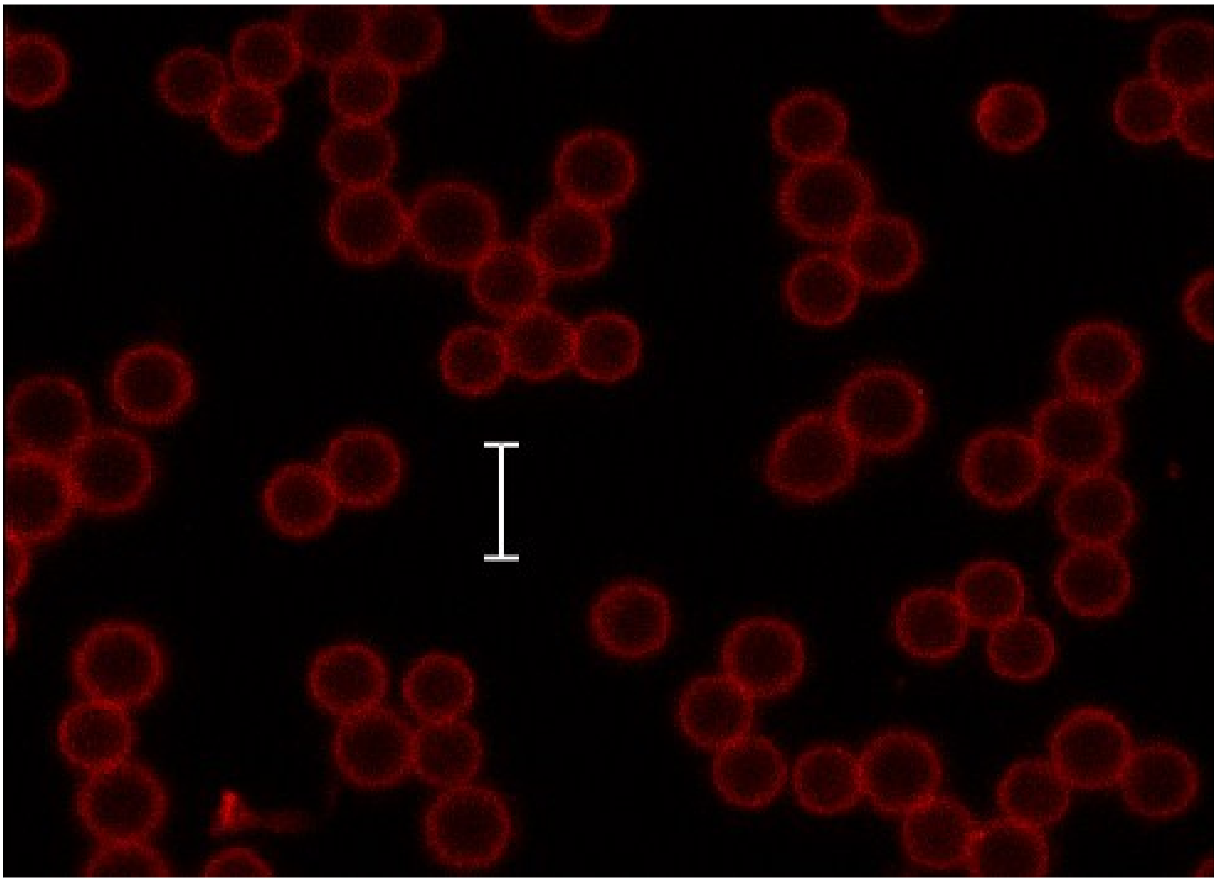

Figure SI5: Representative fluorescence micrograph of loaded glowed, non-porous silica in water with 0.1 mM surfactant **6** and 0.1 M NaOTf electrolyte at the same particle concentration as used in catalysis (50 mg/mL). The length of the scale bar is 10 µm.

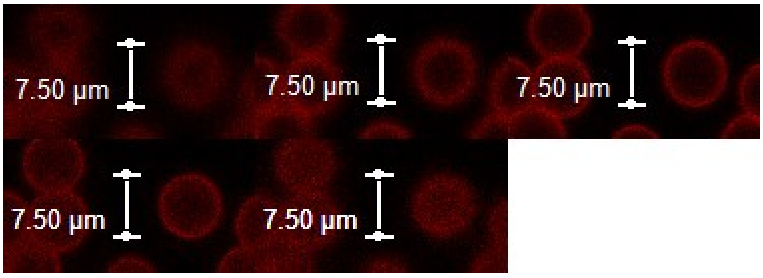

Figure SI6: Representative fluorescence micrograph of a selected non-porous silica particle at different depths (z-direction). The conditions are the same as in Figure SI5 the distance between each picture is 2 µm.

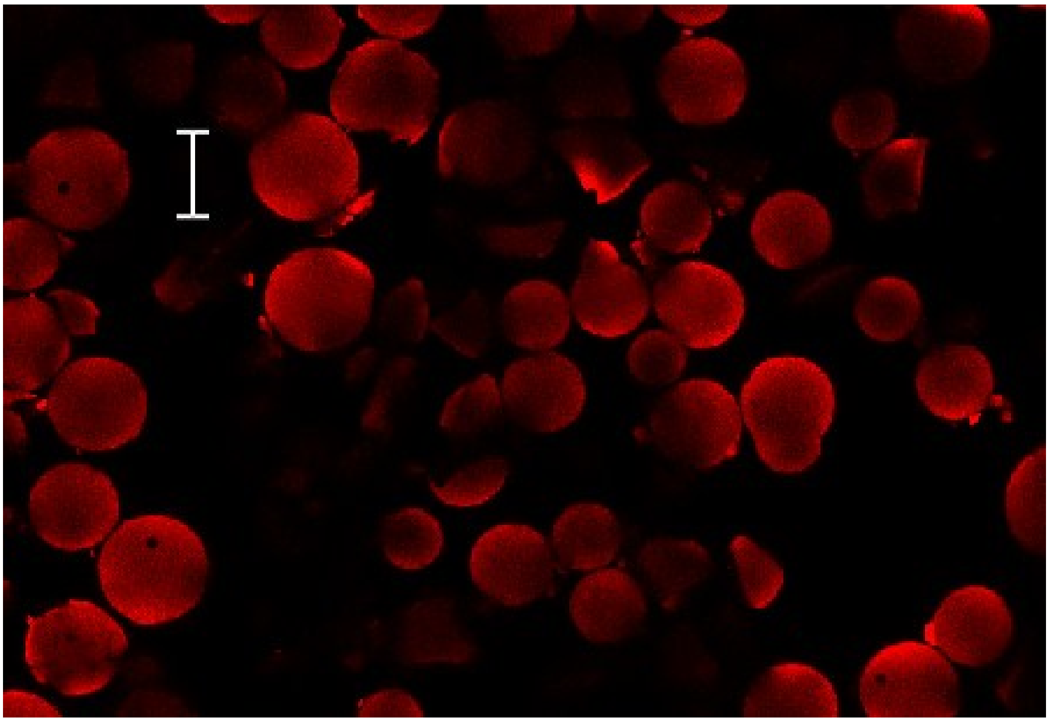

Figure SI7: Representative fluorescence micrograph of loadedporous silica in water with 3 mM surfactant **6** and 0.1 M NaOTf electrolyte at the same particle concentration as used in catalysis (4 mg/mL). The length of the scale bar is 50 µm.

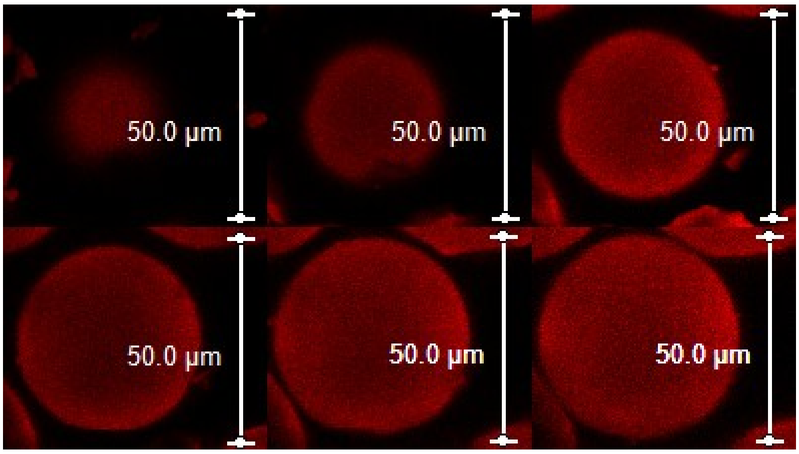

Figure SI8: Representative fluorescence micrograph of a selected porous silica particle at different depths (z-direction). The conditions are the same as in Figure SI7 and the distance between each picture is 5 µm.

6. Additional tables and figures

Table SI2: Maximal rates and total amount of evolved hydrogen for photocatalysis in 10 mL 1 M ascorbate buffer (pH 4) with 0.1 M NaOTf, 200 μM $[Ru(bpy)_3]Cl_2$ and 10 μM **1** with surfactant **6** or **7**, respectively.

Conditions	Max rate (nmol H_2/s)	Total H_2 (μmol)
No surfactant	12 ± 0.4	59.0 ± 4
3 mM **7**	9.4 ± 0.6	56 ± 5
3 mM **6**	11.5± 0.7	36 ± 3

Table SI3: Summarized results for different blank experiments in 10 mL 1 M ascorbate buffer (pH 4) with 0.1 M NaOTf electrolyte.

Conditions	Max rate (nmol H_2/s)	Total H_2 (μmol)
0.5 mM $[Ru(bpy)_3]Cl_2$, no WRC	0.81 ± 0.08	5.3 ± 2.3
0.2 mM PS **5** and 20 μM WRC **3**, on f-SiO_2-C_{18}, 3 mM surfactant **7**. Particles were filtered off and the colorless solution irradiated	<0.01	-
Then 0.5 mM $[Ru(bpy)_3]Cl_2$ was added and irradiation continued	5.2 ± 0.05	3.8 ± 2.6
0.2 mM PS **5** (as dichloride salt), 20 μM WRC **3** and 3 mM **7**; no support; precipitates due to addition of NaOTfls	1.55 ± 0.13	26 ± 6

Table SI4: Maximal rates and total amounts of evolved hydrogen in 1 M ascorbate buffer with 0.1 M NaOTf electrolyte, 0.1 mM PS **5** and 5 µM WRC **3** immobilized on hydrophobic fumed silica at different surface loadings per BET surface area with **7** as surfactant.

([PS] + [WRC])/m^2 (nmol)	Loading molecules/nm^2	f-SiO_2-C_{18} (mg)	[7] (mM)	Max rate (nmol H_2/s)	Total H_2 (µmol)
30	0.018	185	4	1.2 ± 0.11	33 ± 8
75	0.045	75	3	3.3 ± 0.3	45 ± 11
150	0.090	38	2	2.85 ± 0.18	31 ± 3
300	0.181	20	1.5	4 ± 0.3	26 ± 4
450	0.271	12	0.75	3.3± 0.15	21 ± 3

Table SI5: Summarized results of the surfactant dependency study in 1 M ascorbate buffer (pH 4) with 0.2 mM PS **5** and 20 µM WRC **3** immobilized on C_{18}-modified fumed silica and 0.1 M NaOTf at different surfactant concentrations (anionic: Na[C_{12}-PhSO_3], cationic: [C_{16}-NMe_3][OAc]).

[Surfactant] x charge (mM)	Max. rate (nmol H_2/s)	Total H_2 (µmol)
-3.5	0.24 ± 0.04	21.4 ± 6.1
-2.5	1.64 ± 0.12	43.8 ± 9.2
-1.5	2.65 ± 0.18	65.4 ± 10.9
2	3.68 ± 0.24	85.6 ± 9.5
3	3.52 ± 0.23	93.2 ± 10.6
4	3.61 ± 0.24	97.9 ± 11.3

Table SI6: Summarized results of the [WRC] dependency study in 1 M ascorbate buffer (pH 4), 0.1 NaOTf, 0.2 mM **5** and varying [**3**] immobilized on hydrophobic fumed silica.

[WRC] (μM)	Max rate (nmol H_2/s)	Total H_2 (μmol)[a]	TON_{Co} (H_2/Co)[a]
20	4.0 ± 0.6	99.1 ± 10	495 ± 50
10	3.9 ± 0.4	72.1 ± 8	721 ± 80
5	3.9 ± 0.4	66.1 ± 5	1322 ± 101
2	3.2 ± 0.2	25.1 ± 2	1255 ± 106
0.2	0.95 ± 0.06	2.3 ± 0.7	1150 ± 506
0	0.48 ± 0.03	6.9 ± 0.7	-

[a] The amount of H_2 produced in the blank experiment (no WRC) was subtracted

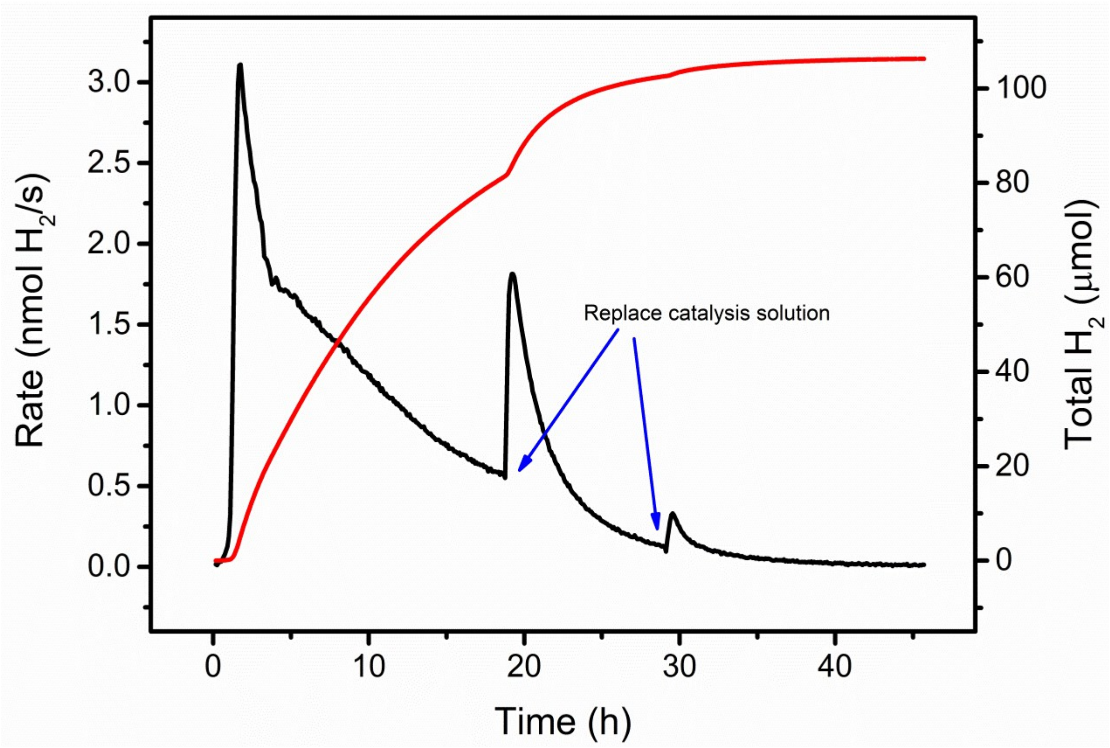

Figure SI9: Rates (**black line**) and amounts of H_2 (**red line**) for a heterogeneous photocatalytic reaction in 1 M ascorbate buffer (pH 4) and 0.1 M NaOTf with 200 μM PS **5** and 40 μM WRC **3** immobilized on f-SiO_2-C_{18} with 3 mM **7**. Blue arrows: Irradiation was stopped, the suspension centrifuged and the particles washed twice with 5 mL 0.1 M aqueous NaOTf. Then, a fresh catalysis solution was added (10 mL 1 M ascorbate buffer, pH 4 with 0.1 NaOTf) and irradiation continued.

7. Transient absorption measurements

Quenching of Ru* with WRC **1** ([CoBr(appy)]Br) was examined in pure water with 100 μM $[Ru(bpy)_3]Cl_2$ and varying concentrations of **1**. No formation of Ru^{III} or Co^{I} was observed, but non-productive quenching of Ru* by **1**. The quench rate was estimated by plotting k_{obs} (1/τ) against the Co concentration (Figure SI3).

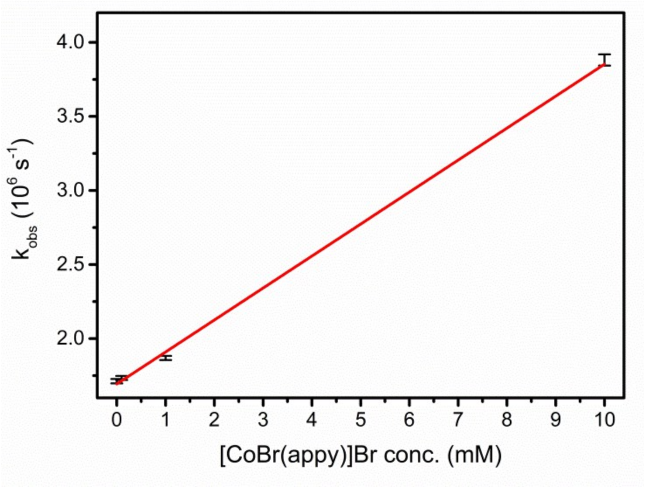

Figure SI10: Determined reaction rates of the quenching study in water with 100 μM $[Ru(bpy)_3]Cl_2$ and 0, 0.1, 1 and 10 mM WRC **1** ([CoBr(appy)]Br).

The quench rate of Ru* by ascorbate was examined in 0, 0.5 and 0.95 M aqueous ascorbate buffer (pH 4) with 50 μM $[Ru(bpy)_3]Cl_2$. Transient absorption spectroscopy clearly showed formation of reduced PS (Figure SI4). Reaction rates (k_{obs}) were obtained by exponential fit of the first 2 components of a SVD analysis. The quench rate was determined by plotting k_{obs} against the ascorbate concentrations (Figure SI5)

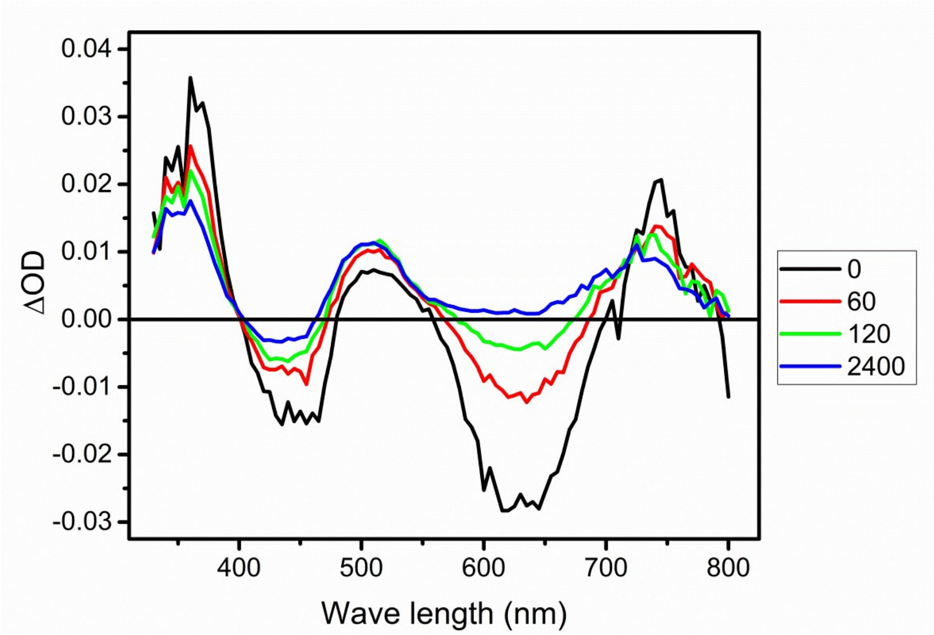

Figure SI11: Transient absorption spectrum of 50 μM $[Ru(bpy)_3]Cl_2$ in 0.95 M ascorbate buffer at selected times (ns) after excitation. The black line represents the transient absorption spectrum of excited Ru (Ru*) and the blue line the one of reduced PS^-.

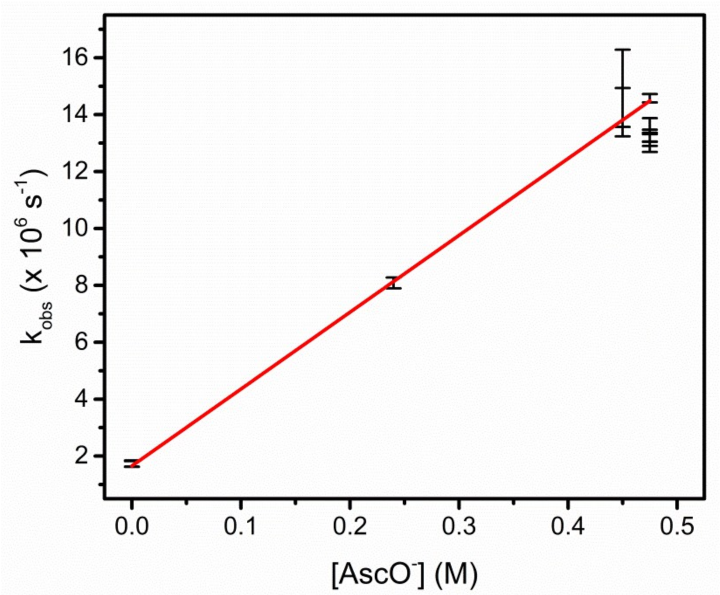

Figure SI12: Determined reaction rates of 50 µM $[Ru(bpy)_3]Cl_2$ in 0, 0.5 and 0.95 M ascorbate buffer.

The electron rate transfer rate from reduced PS to WRC ($PS^- + Co^{II} \rightarrow PS + Co^{I}$) was determined in 0.95 M aqueous ascorbate buffer with 50 µM $[Ru(bpy)_3]Cl_2$ and 0, 100 and 200 µM [CoBr(appy)]Br (**1**). Transient absorption spectroscopy clearly showed formation of PS^- followed by formation of Co^I (Figure SI6). The reaction rates (k_{obs}) were obtained by exponential fit of the first 3 components of a SVD analysis. The electron transfer rate was determined by plotting k_{obs} against the WRC concentrations (Figure SI7).

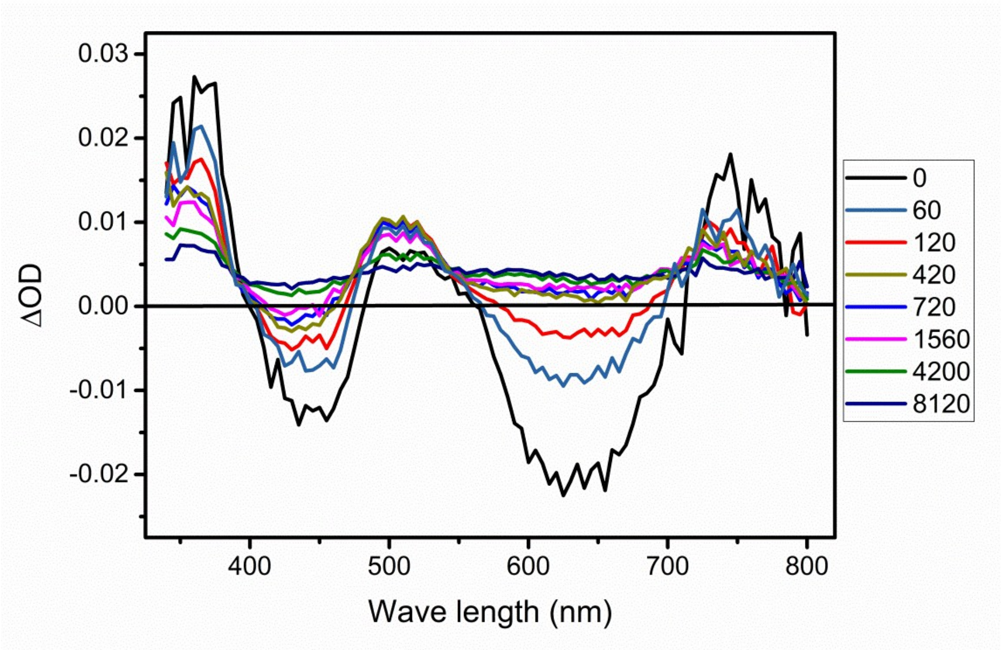

Figure SI13: Transient absorption spectrum of 50 µM $[Ru(bpy)_3]Cl_2$ and 200 µM [CoBr(appy)]Br (**1**) in 0.95 M ascorbate buffer at selected times (ns) after excitation. The dark blue line represents the transient absorption spectrum of reduced WRC ($\mathbf{1}^-$).

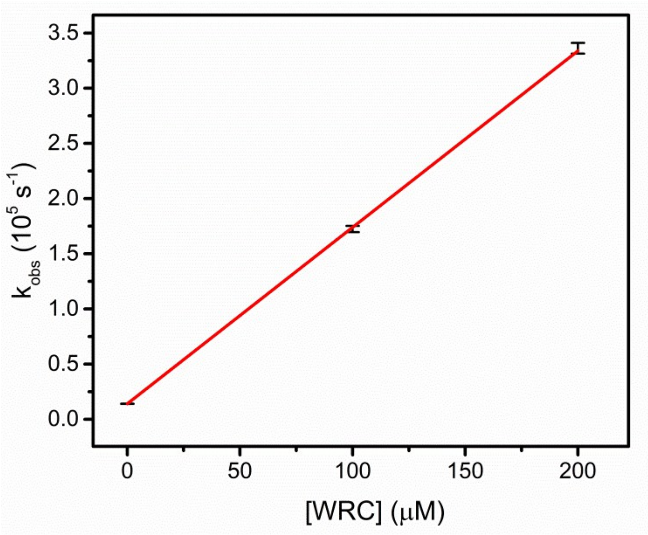

Figure SI14: Determined reaction rates of 50 µM $[Ru(bpy)_3]Cl_2$ in 0.95 M ascorbate buffer (pH 4) and 0,100 and 200 µM WRC **1**.

1. Muller, P.; Brettel, K., *Photochem. Photobiol. Sci.* **2012,** *11*, 632-636.
